# Shaping Fungal Communities in *Cenchrus setaceus*: Host Condition and Habitat Filtering

**DOI:** 10.1007/s00248-026-02805-3

**Published:** 2026-06-16

**Authors:** Andreea Cosoveanu, Mario Andrés González-Carracedo, Jorge Sopena Lasala, José Antonio Pérez Pérez, Raimundo Cabrera

**Affiliations:** 1 Department of Botany, Ecology and Plant Physiology, Universidad de La Laguna, San Cristóbal de La Laguna, Tenerife, 38200 Spain; 2 La Peña Gabinete de Estudios Ambientales, S.L.U.(GEA), El Escobonal, Güimar, Tenerife, Canary Islands 38591 Spain; 3 Department of Biochemistry, Microbiology, Cellular Biology and Genetics, Universidad de La Laguna (ULL), San Cristóbal de La Laguna, Tenerife, 38200 Spain

**Keywords:** Leaf microbiome, Plant-fungal associations, Ecological filtering, Stochastic turnover, MinION nanopore sequencing, Fungal metagenomics

## Abstract

We investigated the leaf-associated fungal communities of *Cenchrus setaceus* across a host condition gradient (high- vs. low-condition plants) and environmental zones (coast vs. hill; trade-wind exposure) on Tenerife (TF) and La Palma (LP). We hypothesized that community assembly reflects both host-driven deterministic filtering and abiotic promotion of richness in favourable environments via two mechanisms: (i) high-condition plants promote stable, guild-structured communities; (ii) humid, topographically buffered zones enhance fungal richness, especially for endophytes and saprotrophs. Nanopore sequencing and functional guild annotation revealed island- and zone-specific fungal assemblages. In TF, low-condition plants were associated with genera linked to stressed or exposed conditions whereas high-condition plants, especially in humid northern hills, supported more recurrent yeast-like and niche-associated taxa. In LP, high-condition plants in eastern hill zones were associated with distinct taxa, while drier western coastal low-condition plants were enriched in stress-related fungi. Fungal genera richness (Hill0) was consistently higher in low-condition plants (TF: 146 vs. 95; LP: 94 vs. 76; *p* < 0.05), while Shannon diversity diverged: greater in high-condition plants on LP (3.29 vs. 2.98), but lower on TF (3.10 vs. 3.28; *p* < 0.05). Community structure was shaped primarily by host condition in TF (PERMANOVA R² = 8.6%, *p* < 0.05), and by zone in LP (R² = 15.0%, *p* < 0.05). On TF, low-condition plants hosted significantly higher richness of saprotrophic, endophytic and plant-pathogenic genera (all *p* ≤ 0.001), whereas in LP zone × condition effects shaped guild richness patterns, with saprotroph richness increasing 2.66-fold in high condition plants from eastern hills relative to the eastern coast. Overall, high-condition plants supported less diverse but compositionally more stable fungal communities, while favourable environments enhanced guild richness independently of host condition.

**Supplementary Information:**

The online version contains supplementary material available at 10.1007/s00248-026-02805-3.

## Introduction

The spread of invasive species poses major threats to biodiversity, particularly in island ecosystems where endemic species often lack evolutionary defences. *Cenchrus setaceus* (Forssk.) Chiov. (syn. *Pennisetum setaceum*) commonly known as fountain grass and locally as “rabo de gato”, exemplifies this threat in the Canary Islands. Since its introduction in 1943, it has become one of the most problematic invasive alien plants in the region [1, 2]. Its invasive success has been documented on numerous islands, including Hawaii, Sardinia, Sicily and Madeira, among others [3–7]. Efforts to control *Cenchrus setaceus* using mechanical or chemical means have largely failed, and no classical biological control agent has yet been implemented [8]. This has stimulated interest in identifying locally occurring fungal taxa associated with the species across gradients of host condition and environment. In this study, we focus on the phyllosphere fungal communities of *C. setaceus*, here defined as fungi associated with leaf tissues, including both epiphytic and endophytic forms. By characterising these communities, we examine how host condition and environmental filtering shape their composition and diversity.

Phyllosphere fungi are taxonomically and functionally diverse, and may influence host performance through effects on leaf senescence, stress responses and disease expression [9–11]. Foliar assemblages include endophytes, epiphytes, saprotrophs and pathogens and their ecological effects may vary with host status and environmental context [12–17]. Community composition is shaped by climatic and microclimatic conditions, particularly humidity, temperature and precipitation, but also by host traits such as leaf area and plant height, which alter exposure to rain, wind and UV radiation [14, 18–21]. Along elevational gradients, even relatively modest environmental shifts may influence fungal richness, guild structure and co-occurrence patterns within a single host species [22]. Comparable patterns have also been reported in grasses, where host identity exerted a stronger influence than elevation on the diversity and composition of above- and belowground fungal endophytes and arbuscular mycorrhizal fungi across replicated altitudinal gradients [23].

From an assembly perspective, phyllosphere communities may reflect both deterministic filtering and stochastic turnover. Host condition may favour recurrent fungal associations, whereas physiologically compromised tissues may allow broader or less predictable colonisation. At the same time, trade-wind exposure and elevation may generate buffered phyllosphere environments, altering opportunities for colonisation across fungal guilds. On oceanic islands, where environmental heterogeneity and dispersal constraints coincide, the relative contribution of these processes remains insufficiently resolved [17, 24–27]. In this context, invasive hosts offer a useful system for examining how biotic and abiotic filters act together, or independently, in shaping foliar fungal communities.

We studied the fungal community associated with *Cenchrus setaceus* across zones that differ in altitude and orientation, and within populations of contrasting plant condition (high versus low, based on morphological and physiological traits) [28]. Specifically, we asked:

i) Does fungal community composition differ between high- and low-condition hosts, or across environmental zones?
ii) Are fungal communities spatially clustered according to host condition or environmental zone?
iii) To what extent are differences in fungal communities driven by random colonisation rather than predictable environmental filters?
iv) How much of the observed variation in fungal composition can be explained by host condition and environmental zone?

We hypothesised that the assembly of plant-associated fungal communities is shaped primarily by deterministic processes driven by both biotic (host condition) and abiotic (environmental) filters. Within this framework, we propose two complementary mechanisms that may operate in parallel. First, host-mediated selection: high-condition plants may promote non-random associations with fungi compatible with sustained growth, particularly endophytes and saprotrophs, leading to more compositionally similar and more clearly guild-structured communities across environmental zones. In contrast, low-condition plants are expected to host more heterogeneous assemblages, with a higher representation of opportunistic and potentially pathogenic taxa and weaker compositional convergence. Second, environmentally facilitated colonisation: favourable environments, particularly more humid zones shaped by trade winds and elevation, may enhance fungal richness, especially for endophytic and saprotrophic guilds. While these groups are expected to increase under humid conditions, pathogen richness may be more context-dependent, reflecting host stress, community interactions and local stochastic effects.

Together, these mechanisms, host-mediated selection and environmentally facilitated colonisation, are hypothesised to contribute jointly to the composition, richness, diversity, and functional structure of fungal communities along gradients of host condition and environmental context.

## Materials and Methods

### Study Site and Plant Sampling

**Fig. 1:**
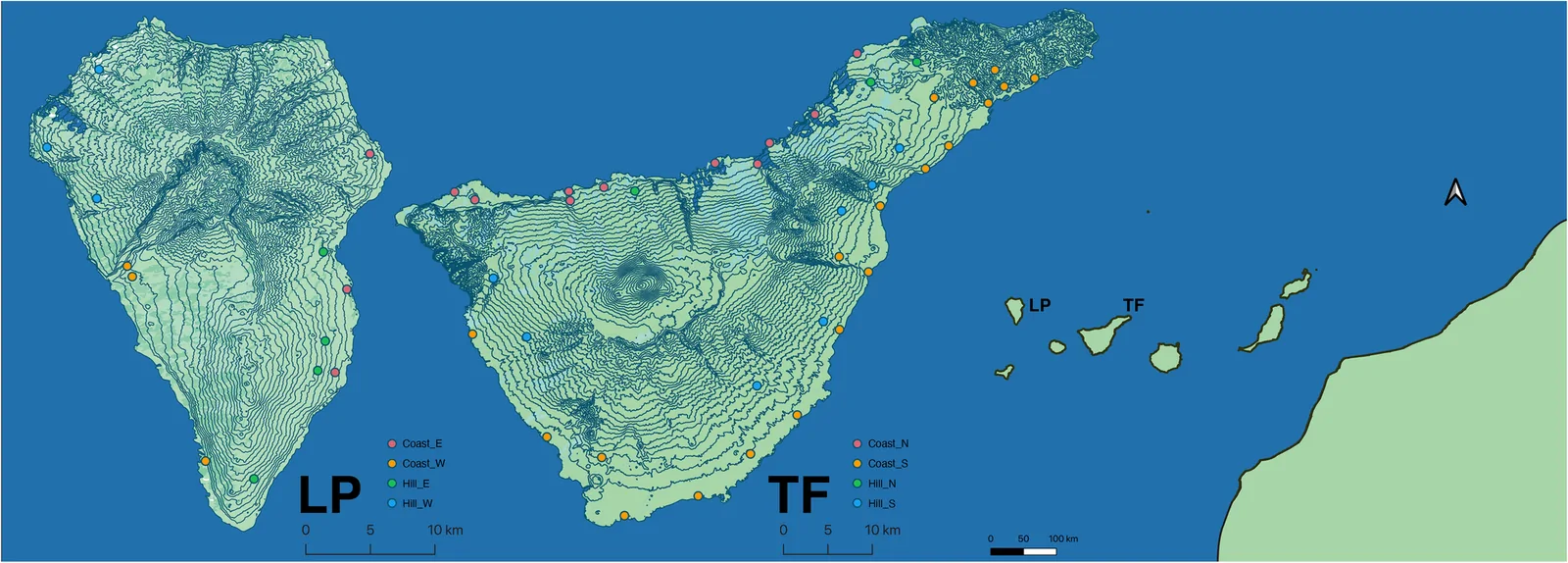
Geographic distribution of sampling sites on La Palma (LP; left; 13 sites) and Tenerife (TF; right; 39 sites), Canary Islands, Spain. Sampling sites are shown as coloured points and are coded according to the four environmental zones used throughout the study, combining elevation class (coast vs. hill) and island orientation (LP: Coast_E, Coast_W, Hill_E, Hill_W; TF: Coast_N, Coast_S, Hill_N, Hill_S). Blue contour lines indicate topography. The inset shows the position of the Canary archipelago relative to the north-west African coastline

Sampling took place from late April to early June 2022 in 39 sites on Tenerife (TF) and 13 sites on La Palma (LP) (Fig. 1). Site selection aimed to capture an altitudinal contrast (coast vs. hill) while keeping replicate sites within the same island in reasonable geographic proximity. In addition, we incorporated the dominant trade-wind exposure gradient on each island, contrasting windward (more humid) versus leeward (drier) sectors. All sites were assigned a priori to environmental zones for downstream analyses (see Statistical analysis).

Environmental zones were defined a priori to represent island-scale bioclimatic structure driven by orography and the trade-wind cloud regime, rather than to optimise separation in the fungal community data. On Tenerife, bioclimatic reconstructions show higher humidity and rainfall on northern slopes, particularly in areas receiving frequent fog-water inputs associated with trade-wind clouds, and comparatively drier conditions on southern slopes [29]. On La Palma, the trade-wind regime generates cloud formation on the northern and northeastern sectors, whereas the remaining sectors experience substantially reduced cloud influence, producing a marked climatic partition at island scale [30]. Altitudinal class was defined using a fixed elevation cut-off of 300 m a.s.l.; sites < 300 m classified as coast, and sites ≥ 300 m as hill. These exposure × altitude combinations yielded four environmental zones per island (TF: north coast, north hill, south coast, south hill; LP: east coast, east hill, west coast, west hill). This zoning is consistent with bioclimatic and vegetation syntheses showing that trade-wind cloud influence and humidity gradients interact with elevation to structure ombrotype distributions and vegetation belts on both islands [29–35].

Using UTM GPS coordinates for the six sampled plants per site, we quantified site footprint as convex-hull area and within-site spacing as nearest-neighbour and maximum inter-plant distances. Median site area was 258 m^2^ (range 9.24–2976) in Tenerife (39 sites) and 488 m^2^ (range 69.6–1419) in La Palma (13 sites). Median nearest-neighbour distance was 6.91 m (range 1.07–16.5) in Tenerife and 7.63 m (range 1.41–14.1) in La Palma, while median maximum inter-plant distance was 38.4 m (range 7.28–159) and 57.6 m (range 38.1–120), respectively (Table S6).

All community analyses were conducted separately for each island because TF and LP differ in their climatic space, vegetation mosaics, and regional species pools, so pooling islands would conflate island identity with environmental effects (see Statistical analysis).

Plants were assigned to two host condition classes (hereafter high-condition and low-condition plants) in the field, based on whole-plant foliar status (> 90% foliage in the same state across the crown) together with the reproductive output (inflorescence abundance). High-condition plants were identified by i) > 90% healthy green foliage and ii) abundant inflorescence production, whereas low-condition plants showed i) > 90% chlorotic, dry, or necrotic foliage and ii) low inflorescence production. At sampling we recorded biometric and reproductive traits (plant height, two crown diameters at the top of the plant and basal perimeter and inflorescence count). This condition gradient was previously evaluated in a separate dataset using Kruskal-Wallis tests, Spearman rank correlations and regression analyses between plant volume and inflorescence number within each condition class and island, which consistently distinguished high- from low- condition plants [28]. At each site, six plants were sampled: three high-condition and three low-condition individuals. For each plant, three groups of leaves were cut with sterilized pruning shears, and five leaves were randomly selected for analysis. Asymptomatic green leaves were sampled from high-condition plants, while chlorotic leaves were selected from low-condition individuals. Dark grey leaves were excluded due to saprotroph colonisation of senescent tissue. Because leaf tissue state is intrinsically linked to the field-assigned host-condition classes, our sampling contrasts plant condition together with its associated leaf-state phenotype (green functional tissue in high-condition plants versus chlorotic/dry tissue in low-condition plants), and their effects are not fully separable in this design.

Samples were placed in sterile zip-lock bags and stored in a cooler bag, until lab transfer. Scissors and gloves were disinfected between plants using 15% bleach and 70% ethanol to prevent DNA cross-contamination. One zip-lock bag containing sterile paper was opened at each site and handled in the same way as the bags used for leaf collection; the paper was the retained and processed as an environmental control sample. All leaf samples and environmental controls were processed within 48 h.

### Plant Sample Processing and DNA Extraction

Low condition leaves were typically chlorotic and partially desiccated, whereas high-condition leaves were hydrated. In preliminary trials we therefore standardised sampling primarily by comparable leaf length/area and used mass as the practical proxy compatible with extraction tube volume and kit specifications. Repeated weighing of comparable leaf lengths indicated an approximate 1:2 mass ratio between chlorotic/desiccated and hydrated/healthy tissue. We tested two extraction kits and several input-mass combinations (including 30/60 mg and 50/100 mg) and selected 30 mg (low condition) and 60 mg (high condition) as the most reproducible combination for DNA yield and downstream amplification while approximating comparable leaf-area input across host-condition categories.

To minimise downstream bias introduced by extraction yield heterogeneity, we applied the same processing steps across all samples. Leaf blades were trimmed to exclude tips and sheaths (the first 1–2 cm discarded), fragments (~ 0.5 cm) were homogenised under identical conditions, and only chlorotic tissue (low condition) or only healthy green tissue (high condition) was used to avoid mixing tissue states within a sample. Subsamples (30 mg or 60 mg, as above) were transferred into lysing matrix microtubes (FastPrep-24™ Classic, MP Biomedicals), flash-frozen in liquid nitrogen, and stored at −80 °C.

Genomic DNA was extracted using the E.Z.N.A. Plant DNA DS kit (Omega Bio-tek), following the manufacturer’s recommendations. DNA was quantified using a Qubit 4 fluorometer (Thermo Fisher Scientific), with the Broad Range (BR) Assay Kit (Thermo Fisher Scientific), according to the manufacturer’s instructions, and stored at −20 °C until use. No separate negative extraction blanks were included in the sequencing runs; instead, potential contamination associated with extraction and subsequent steps was monitored through the environmental control samples (see Study site and plant sampling) and the PCR negative controls described below.

### Primer Design, Testing and qPCR

Primers ITS1Fngs and ITS4Fun, which amplify the complete fungal ITS1–5.8S – ITS2 region, were adopted from Nilsson et al. [36]. Primer ITS4Fun was shortened at both its 3’ and 5’ ends to lower its melting temperature and reduce stable secondary structures and was renamed ITS4Funy. Both primers were 5’-extended with the tails specified by EXP-PBC096 PCR Barcoding Expansion 1–96 Kit (ONT, UK). The final primer sequences were 5’- TTTCTGTTGGTGCTGATATTGCGGTCATTTAGAGGAAGTAA − 3’ (ITS1Fngs) and 5’- ACTTGCCTGTCGCTCTATCTTCCCTCCGCTTATTGATATGCT − 3’ (ITS4Funy). Prior to library preparation, four primer combinations were evaluated by qPCR using genomic DNA from two *C. setaceus* leaf samples (chlorotic/dry tissue for low-condition plants and green/functional tissue for high-condition plants) and from a representative foliar fungal isolate. Template DNA was standardised to 1 ng µl^− 1^. Reactions (20 µl) contained 10 µl of 2× PerfeCTa SYBR Green FastMix (Quantabio), 2 µl of each primer (2 µM), and 6 µl of template DNA (1 ng µl^− 1^); negative controls contained H_2_O instead of template DNA. qPCRs were run on a LightCycler 480 Instrument (Roche Diagnostics), with one cycle of 95 °C for 2 min, followed by 45 cycles of 95 °C for 15 s, 50 °C for 10 s, and 72 °C for 90 s. threshold cycle (Ct) values were determined automatically by the instrument software. Primer performance and specificity were assessed by comparing amplification profiles across plant DNA extracts, fungal DNA, and no-template controls. Based on these qPCR tests, the primer pair ITS1Fngs/ITS4Funy was selected for subsequent amplification of fungal ITS from *C. setaceus* samples.

The same qPCR conditions were then used to estimate the optimal cycle number for the first PCR during library preparation. DNA preparations from *C. setaceus* were diluted to 1 ng µl^− 1^ and tested in duplicate. Ct values ranged from 13 to 34 and samples were grouped into seven strata of three Ct units. For each stratum, the cycle number for the first PCR was set as the upper Ct bound plus three cycles, so that samples were amplified to comparable yields while remaining within the exponential phase.

### Mock Community Preparation

Six fungal isolates were selected for the preparation of a mock community adapted to this study: *Aplosporella prunicola*, *Fusarium graminearum* and *Fusarium clavum* previously isolated in our laboratory from *C. setaceus*, as well as the fungal pathogens *Fusarium oxysporum*, *Alternaria brassicicola* and *Botrytis cinerea*. Genomic DNA was purified and quantified as previously described. Identical volumes of the six genomic DNA extracts at 1 ng µl^− 1^ were mixed to obtain the mock community. Aliquots of this mock community were included as technical control wells on each MinION sequencing plate and processed in parallel with field samples, to confirm that all expected genera could be recovered by the pipeline and to evaluate distortions in relative abundance introduced by amplification, sequencing, and clustering (Table S1).

## Preparation of Sequencing Libraries

The sequencing libraries were prepared following the procedures recommended for the SQK-LSK110 Ligation Sequencing Kit (ONT, UK), and EXP-PBC-096 PCR Barcoding Expansion 1–96 Kit (ONT, UK). First round PCRs contains 2 µl of 5X Phusion HF Buffer (Thermo Fisher Scientific), 2 µl of dNTP mix (2 mM each), 2 µl of primers ITS1Fngs and ITS1Funy (2 µM each), 0.2 µl of Phusion High-Fidelity DNA Polymerase (Thermo Fisher Scientific), 6 µl of template DNA (1 ng µl^− 1^) or non-diluted controls, and H_2_O to a final volume of 20 µl. PCRs were run on a ProFlex PCR system (Applied Biosystems), including one cycle of 98 °C for 30 s, followed by a sample-specific number of cycles (19, 22, 25, 28, 31, 34 or 37 cycles, as previously determined by qPCR) of 98 °C for 10 s, 50 °C for 10 s, and 72 °C for 90 s. A final extension step of 72 °C for 15 s was included. Environmental control samples (*n* = 32, consisting of DNA extracted from the exposed sterile paper described above) and PCR negative controls (no template; *n* = 2) were amplified at 37 cycles and processed in parallel with the leaf samples. A representative set of samples (*n* = 64) was selected for their analysis in 2% agarose gel. A semiquantitative value for the expected average concentration of all PCR products was estimated from the gels by densitometry, using Gel Analyzer 19.1 software, and the 100 bp DNA ladder (VWR) as reference. PCR products were enzymatically cleaned by mixing 5 µl of each PCR product with 1.5 µl of ExoCleanUp reagent (VWR). Samples were incubated at 37 °C for 30 min, and 85 °C for 15 min, and then diluted to 0.5 nM with 10 mM Tris-HCl pH 8.0, assuming an average length of 700 bp for all PCR products to estimate molar concentration. Samples were distributed in four 96-well plates, to prepare the indexing PCRs in four sequencing runs. Each plate contains two mock community replicates, the environmental control samples corresponding to the field samples on that plate, and PCR negative controls (no-template), all processed and sequenced together with the leaf samples. Indexing PCR reactions contain 6 µl of a 0.5 nM PCR product, 4 µl of 1 µM DNA-Barcode pair from EXP-PBC096 kit (ONT), and 10 µl of 2X LongAmp Taq Master Mix (NEB, USA). Thermal cycling program includes one initial denaturation step (95º C, 3 min), 12 cycles of denaturation (95 °C, 15 s), annealing (62 °C, 15 s), and extension (65 °C, 1.5 min), and a final extension step (65 °C, 7 min). A subset of indexed PCR products (*n* = 64) was subjected to agarose gel electrophoresis for analysis, and mean concentration was estimated by densitometry. Within each 96-well plate, equal volumes of purified amplicons from all wells were pooled, and DNA was purified with the Agencourt AMPure XP Beads (Beckman Coulter, USA), following the manufacturer’s instructions. The pooled library for each plate was quantified by spectrophotometry, diluted to a final concentration of 25 ng µl^− 1^ with H_2_O, and stored at −20 °C until use. Forty-seven microlitres of each pooled library (25 ng µl^− 1^, ~ 1.2 µg DNA) were then mixed with 1 µl of DNA CS (ONT, UK), 3.5 µl of NEBNext FFPE DNA Repair Buffer (NEB, USA), 2 µl of NEBNext FFPE DNA Repair Buffer (NEB, USA), 3.5 µl of NEBNext Ultra II End Prep Reaction Buffer (NEB, USA) and 3 µl of NEBNext Ultra II End Prep Enzyme Mix (NEB, USA). Samples were incubated (20 °C, 5 min), and enzymes were inactivated (65 °C, 5 min). Each sample was purified with Agencourt AMPure XP Beads (Beckman Coulter, USA), following manufacturer’s instructions, and subjected to spectrophotometry quantification. For binding of ONT sequencing adapters, 60 µl of the library were mixed with 25 µl of Ligation Buffer (ONT, UK), 10 µl of NEBNext Quick T4 DNA Ligase (NEB, USA), and 5 µl of Adapter Mix F (ONT, UK). Sample was incubated (20 °C, 10 min), and purified by adding 40 µl of Agencourt AMPure XP Beads (Beckman Coulters, USA). Beads were washed twice with 250 µl of Short Fragment Buffer (ONT, UK), and the purified library was eluted in 15 µl of Elution Buffer (ONT, UK). Library was quantified by spectrophotometry, diluted to a final concentration of ~ 4.2 fmol/µl (18.6 ng µl^− 1^), considering an average length of 700 bp for all amplicons with Elution Buffer (ONT, UK), and stored at 4 °C until loading for a maximum of 24 h.

The number of active pores in the FLO-MIN106D (R9.4.1) flow cell (ONT, UK) was checked with the MinKNOW V.22.03.04 software (ONT, UK), in a MinION-Mk1C (ONT, UK) sequencing device. Flow cell was primed, and sequencing library was prepared for loading in the flow cell by mixing 37.5 µl of Sequencing Buffer II (ONT, UK), 25.5 µl of Loading Beads II (ONT, UK), and 12 µl of the DNA library (4.2 fmol µl^− 1^). Sequencing run was carried out for 24 h. Live-basecalling and demultiplexing options were not selected.

### DNA Sequencing and Bioinformatics

Basecalling was conducted post-run on FAST5 files using Guppy v6.0.6 (Oxford Nanopore Technologies) in fast mode. Reads were demultiplexed in MinKNOW (Oxford Nanopore Technologies) using the barcode kit settings, requiring concordant barcode detection on both ends. Quality filtering retained reads with mean Q-score ≥ 8 at the basecalling stage. Run-level read-length and quality distributions for demultiplexed barcode-assigned reads were summarised with NanoPlot/NanoStats (Table S2), following De Coster et al. [37] reporting total reads, total bases, read-length summary statistics, and the proportion of reads with Q-scores above 7, 10, 12 and 15. Across the four MinION plates, mean read length ranged between 598 and 657 bp (medians 586–694 bp), with most reads falling within the expected range for the ITS1-5.8 S-ITS2 region in the sampled fungal taxa. Demultiplexed FASTQ reads were concatenated per barcode and analysed using the SCATA platform (web version as of July 2023) [38].

Filtering and clustering were performed in SCATA using USEARCH as clustering engine [39]. Primer matching was set to ≥ 50% alignment to the primer sequence. SCATA read filtering was performed with minimum mean base quality = 1 and minimum single-base quality = 1; chimera detection and amplicon-level quality control were enabled, and reads shorter than 200 bp were discarded. Sequences were clustered into operational taxonomic units (OTUs) using a maximum pairwise divergence of 0.015 (1.5%) and a minimum alignment coverage of 0.85 of the longer sequence. Homopolymers longer than three bases were collapsed before clustering, while mismatch and gap penalties were kept at their default values (mismatch penalty = 1, gap-open penalty = 0, gap-extension penalty = 1; end-gap weight = 0). For each barcode, we extracted from the SCATA clustering reports the number of reads passing QC after filtering and summarised their distribution across samples to describe effective sequencing depth. After SCATA filtering, the number of reads passing QC per sample ranged from 1 to 105,522 (median 13,479 reads across 379 samples). Within each plate, we report the per-sample distribution (minimum, median and maximum) for demultiplexed reads and reads passing SCATA quality control across all barcode-assigned samples processed in that run (Table S3). To document the subset that entered downstream ecological analyses, we additionally summarised SCATA yield and taxonomic assignment success only for barcode runs and representative sequences retained in the curated genus-level workflow after taxonomic filtering and sample-level curation for the ecological workflow (for instance, no representative sequences available for downstream taxonomic curation; Tables S4 and S5). Thus, Tables S4 and S5 do not represent the full set of 379 barcode-assigned samples reported in Table S3, but the curated subset used to construct the ecological community matrices.

For each OTU, the representative sequence was compared against the UNITE reference database (July 2023 release) using BLASTn [40]. Representative sequences were also compared against fungal ITS reference sequences from type material in the NCBI database using the megablast algorithm. BLAST output was parsed using custom Python scripts (v3.11.4) to extract sample and cluster IDs, taxon names, and similarity statistics. Taxonomic ranks were assigned using overlap and identity thresholds for genus (95–98% overlap and > 94% identity), and species (95–98% overlap and > 99% identity), following Vu et al. [41]. Higher-level classifications were verified using Lifemap NCBI and MycoBank. OTUs whose representative sequences met the quality, length, and taxonomic criteria, including a confident genus-level assignment were retained for subsequent ecological analyses. Summary statistics on annotation success at genus level (and the proportion of unassigned OTUs) and yield by island are reported per island (Tables S4, S5). We therefore report the number of genus-level records retained before and after matrix-level curation (host condition: high – 3948 to 2248; low – 8729 to 8425), to document the magnitude of post-annotation cleaning, which is distinct from read-level QC and OTU clustering. Throughout the manuscript, “reads” refers to demultiplexed barcode-assigned MinION sequences (before clustering), “OTUs” refers to SCATA sequence clusters, and “genera” refers to taxonomic groupings obtained by aggregating OTUs sharing the same genus label. In ecological community matrices and rarefaction analyses, rarefaction was computed from the genus-level count matrix derived from SCATA read counts after filtering and taxonomic curation. Raw reads were deposited in the NCBI Sequence Read Archive under accession number: PRJNA1294559.

### Statistical Analysis

All statistical analyses described below were conducted separately for Tenerife (TF) and La Palma (LP). The representation of environmental stratification (zones and orientations), as well as the total number of sampled plants, were not strictly identical between islands. A single combined analysis would thus confound island identity with environmental factors and produce an unbalanced design. By analysing each island separately, we evaluated the effects of host condition (high versus low) and local environment on fungal communities within each biogeographical unit and then compared patterns qualitatively between islands. Environmental zone contrasts should be viewed as broad-scale associations across spatially structured sites rather than fully independent replicated “humid vs dry” treatments (Table S6). Plant metadata and grouping variables are shown in Table S7.

For each island, we generated genus-level community tables in two representations: (i) a plant × genus presence-absence matrix for prevalence-based summaries and indicator analyses, and (ii) plant-level genus-count matrices for richness/diversity summaries; community-composition analyses were performed using Bray-Curtis dissimilarities computed from genus-level presence-absence tables (binary; Sorensen-type), as detailed below. In both representations, only OTUs with a confirmed genus-level assignment were retained, and OTUs sharing a genus label were pooled so that each column represents one fungal genus. Each site corresponded to a sampling location with six plants (three low-condition and three high-condition), and sites were grouped into four environmental zones per island based on exposure (North/South for TF; East/West for LP) and altitude (coast/hill).

For each site, the frequency of occurrence of each genus was calculated as the proportion of sampled plants in which that genus was detected (number of positive plants/total plants at the site). Genera with non-zero frequency of occurrence in at least one site were retained for downstream analyses and are referred to here as retained genera (i.e. genera detected in at least one plant within at least one site); genera absent from all samples were removed.

For each zone, mean frequencies of occurrence of retained genera were computed across sites and visualised in Circos plots (“circlize” package) [42], where link width reflects mean frequency of occurrence and chord colours represent zones. Circos plots display only genus × zone combinations with mean frequency > 5%, to focus on recurrent genera and keep diagrams interpretable.

Retained genera were also summarised across zone × host condition groups (high- and low-condition plants within each zone). Within each group, counts of plants colonised by each retained genus were converted to relative frequencies of occurrence (proportions summing to 1 per group). For graphical clarity in stacked bar plots, genera contributing < 2% of the total relative frequency in TF or < 3% in LP were grouped; these thresholds were applied only for visual aggregation and were not used to truncate the datasets used in formal analyses. Stacked bar plots were generated using “ggplot2” package [43].

Indicator species analysis (ISA) [44] was performed separately for each island on plant × genus presence-absence matrices including all retained genera. ISA was implemented with the *multipatt()* function (IndVal.g method, 999 permutations, duleg = TRUE) in the “indicspecies” package [45]. Zone × host condition combinations were used as grouping levels. Interpretation was based on the two components of the indicator value, specificity A and fidelity B, together with significance (α = 0.05). Only genera with A or B ≥ 0.7, IndVal.g > 0.5, and *p* < 0.05 were retained as strong indicators. Group sample sizes were checked prior interpretation. No multiple testing correction was applied.

Functional guilds were assigned to all retained genera using the FUNGuild database [46]. Only highly probable assignments were retained, and the primary guild assignment for each genus was used. Guilds were grouped into endophyte, saprotroph, plant pathogen, other pathotrophs, and ectomycorrhizal categories. Genera without a highly probable assignment or lacking one, were classified as Unassigned for visualisation. Guild composition was then visualised for each zone × host condition group using faceted pie charts in the “ggplot2” package, based on the full retained genus matrix prior to any graphical aggregation.

Rarefaction was computed at genus level from site × host condition pooled count tables, obtained by summing genus counts across the three plants of the same host-condition within each site. To avoid unstable curves driven by extremely sparse pooled groups, site × host condition groups with < 10 total genus-assigned OTU records or < 2 observed genera were excluded; the retained groups are reported in Table S8. Rarefaction curves were generated with *rarecurve* function in the “vegan” package [47], and plotted separately for Tenerife and La Palma, with curves coloured by host condition. Because sequencing depth differed among pooled groups, rarefaction is presented descriptively (curve shape/coverage) rather than as a basis for single-depth normalisation.

Alpha diversity was assessed using two indices: Shannon, and Hill0 (richness), calculated at plant level from unrarefied genus-level abundance data with the *diversity* and *specnumber* functions from the “vegan” package. To evaluate the effects of host condition, environmental zone, and their interaction, three grouping variables were used: host condition (low-, high-), zone (altitude = coast vs. hill and orientation N/S and E/W for TF and LP, respectively) and host condition × zone. Depending on the distribution either one-way analysis of variance (ANOVA) or Kruskal-Wallis tests were applied. When global tests were significant, pairwise differences were evaluated using Tukey’s HSD (after ANOVA) or Dunn test with Bonferroni correction (after Kruskal-Wallis), using the “stats” [48], “FSA” [49] and “rstatix” [50] packages.

Permutational multivariate analysis of variance (PERMANOVA) was used to assess the influence of host condition (high vs. low), environmental zone, and their interaction on fungal community composition. For each island, analyses were based on Bray-Curtis dissimilarities computed from plant × genus abundance matrices constructed after filtering genera by site-level prevalence, retaining genera present in at least one plant within at least one location and preserving the location × genus prevalence filter used in the analytical pipeline (> 0.01, where prevalence = number of plants with the genus/number of plants sampled at that location). Environmental zone was defined as a four-level factor combining exposure and altitude (TF: north coast, north hill, south coast, south hill; LP: east coast, east hill, west coast, west hill), and individual plants were treated as sampling units. For each island, PERMANOVA models had the form Bray-Curtis distance ~ host-condition × zone, and were fitted with the *adonis2* function in the “vegan” package [51] using 999 unrestricted permutations with sequentially added terms. Permutation-based inference was handled with support from the “permute” package [52]. For each factor and the interaction term we report pseudo-F, degrees of freedom (Df), R^2^ and permutation-based p-values. The final matrices used for PERMANOVA contained 204 plants × 184 retained genera for TF and 69 plants × 126 retained genera for LP.

Compositional similarity was then tested with Mantel tests based on Pearson correlations with 999 permutations [53] between these community dissimilarity matrices and environmental distance matrices encoding (i) zone × host condition, (ii) zone and (iii) host condition, using the *mantel* function in “vegan”. Additional Mantel tests were performed using pairwise geographic distance matrices calculated from plant UTM coordinates, to evaluate whether fungal community dissimilarity increased with spatial separation independently of the categorical environmental grouping variables.

Non-metric multidimensional scaling (NMDS) ordinations were performed separately for each island using *metaMDS* (“vegan”) on Bray-Curtis dissimilarities derived from the same plant × genus abundance matrices used for PERMANOVA, with two dimensions (k = 2) and 100 iterations (trymax = 100), and automatic transformation enabled [47]. To avoid distortion by extreme single-genus samples, one outlier was removed per island prior to ordination (MTF18E2 for TF and MLP13S2 for LP). Stress values were inspected to confirm convergence and interpretability of the two-dimensional solutions, and ordination scores were examined for range and consistency. Convex hulls were then plotted for host condition × zone groups represented by at least three samples.

To assess fungal community assembly mechanisms, we computed pairwise Raup-Crick dissimilarities (RC_bray) from presence-absence versions of the same retained plant × genus matrices. Abundance matrices were converted to binary format using *decostand* (method = “pa”), and empty rows or columns, if present, were removed before analysis. A custom function adapted from Stegen et al. [54] was used with 999 null iterations. For each sample pair, the observed number of shared genera was compared with a null distribution generated by randomly drawing genera without replacement from the island-specific genus pool while preserving the observed genus richness of each sample. Resulting scores were standardised to range from − 1 to 1. Following the Stegen framework, values > 0.95 were interpreted as heterogeneous selection, values < −0.95 as homogenous selection, and intermediate values as consistent with stochastic assembly. Pairwise RC_bray were reshaped into long format and annotated with host-condition and zone metadata to summarise within- and between-group assembly patterns.

To quantify the relative contributions of host condition and environmental structure to fungal community composition, variation partitioning analysis (VPA) was performed separately for each island using *varpart* function in the “vegan” package [55]. The response matrix consisted of the retained plant × genus abundance matrix used in PERMANOVA and NMDS, converted to numeric format. Two explanatory components were considered: host condition and environmental zone. Explanatory matrices were derived from aligned sample metadata, and all matrices were checked to preserve row-wise correspondence between community data and predictors before partitioning.

To test how fungal guild richness responded to host condition and environmental factors, we used generalised linear mixed models (GLMMs) fitted to richness counts of saprotrophs, endophytes and plant pathogens per plant. Guilds were assigned to retained genera (as defined above). Models were fitted using *glmer* or *glmer.nb* functions under “lme4” package, with Poisson or negative binomial errors depending on dispersion [56]. Overdispersion was assessed using Pearson residuals; a ratio > 1.2 and *p* < 0.05 were used to confirm it. Distributions were inspected visually and statistically for skewness, sparsity, and zero inflation. Model comparisons were performed using likelihood ratio tests via *anova* and AIC [57]. Model selection prioritised parsimony, statistical support (ΔAIC > 2; *p* < 0.05) and biological interpretability. For saprotrophs (TF and LP) and endophytes (LP), the interaction model improved fit (ΔAIC > 3; *p* < 0.05), even if individual interaction terms were not always significant. For pathogens, models excluded interactions (*p* > 0.65). Exponentiated model coefficients were used to estimate richness shifts relative to a baseline group (TF: high-condition in the northern coast; LP: high-condition in the eastern coast). For all guild-richness models, sampling location was included as a random intercept to account for non-independence among plants collected within the same site.

**Fig. 2:**
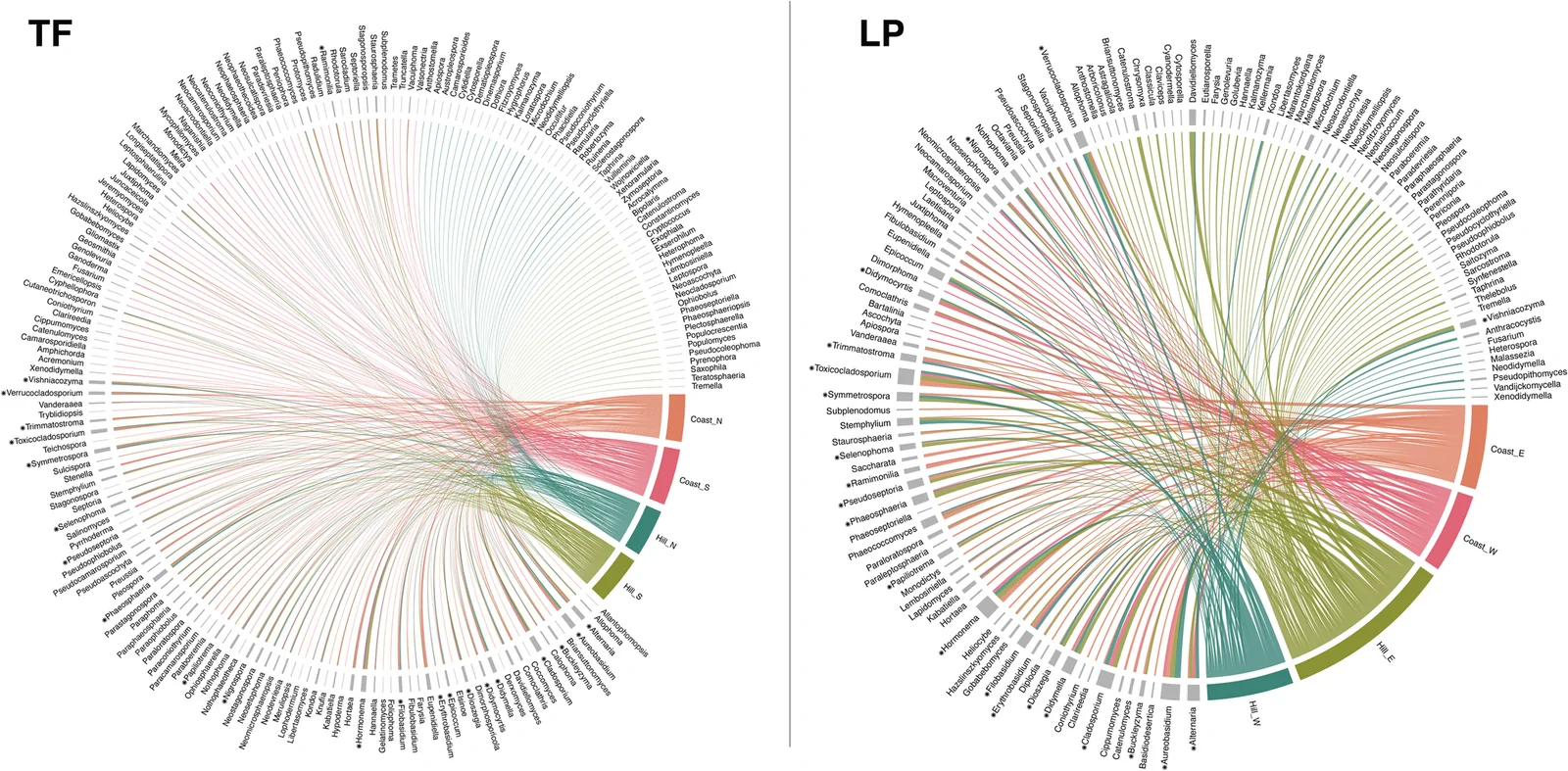
Circos plots showing retained genera detected in TF (left) and LP (right) with mean frequencies of occurrence > 5% per zone (see Methods). Links are colour-coded by zone and connected to genera displayed in neutral grey tones; genera are ordered alphabetically within each island plot. Genera marked with the symbol “⁕” were detected in both islands among the genera displayed in the plots. Link width is proportional to the mean frequency of occurrence of each genus within a zone. Grey inner rectangles are proportional to the summed mean frequency of all displayed genera in each zone and represent total contribution per zone

**Fig. 3:**
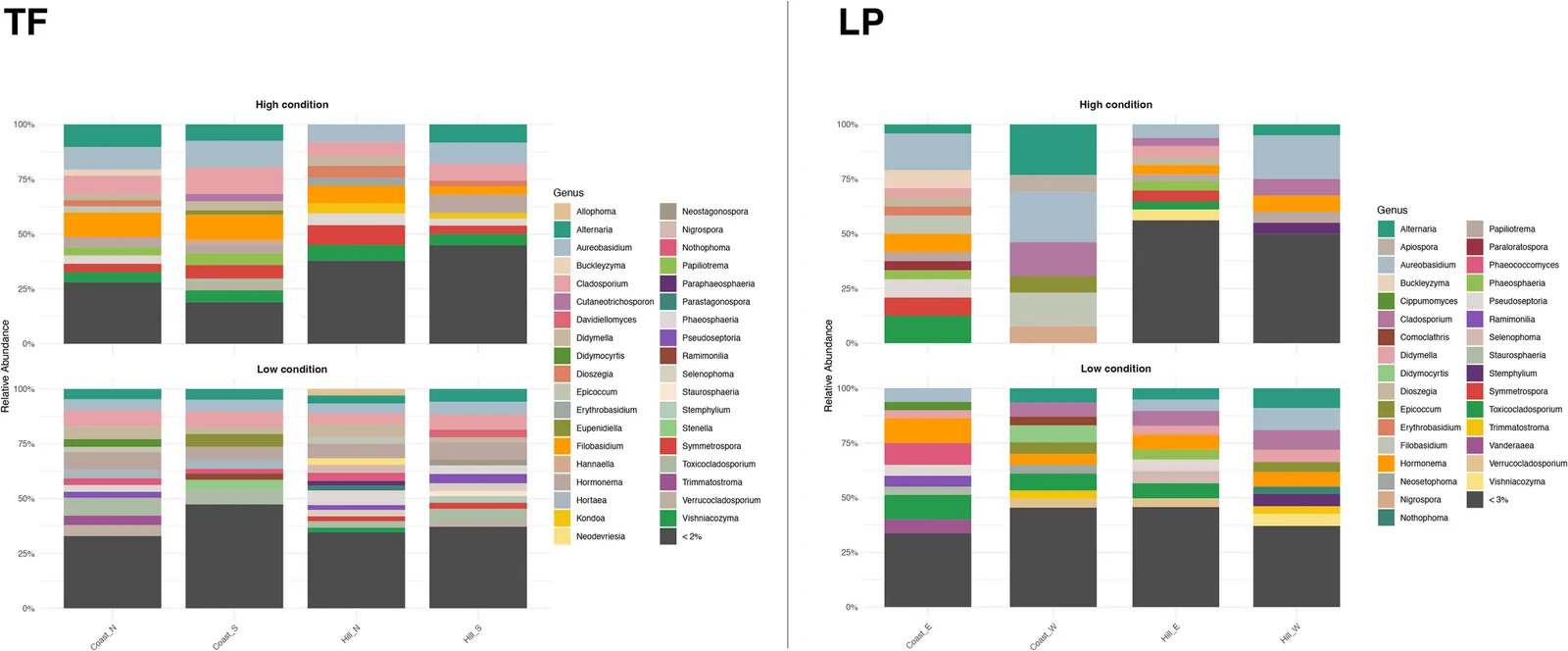
Stacked barplots showing relative frequencies of occurrence of retained fungal genera in TF (left) and LP (right). Retained genera were defined by a frequency of occurrence threshold of 1% per site (i.e. present in at least one of the six plants sampled at a site). Within each zone × host-condition group, relative frequencies of occurrence were normalised to sum 1. For graphical clarity, genera contributing < 2% of the total relative frequency in TF and < 3% in LP were grouped into the categories “< 2%” and “< 3%”, respectively. These thresholds were applied only for visual aggregation of low-frequency genera and were not used in the formal community analyses

To make data provenance explicit, each figure and statistical test above is tied to a defined community table and aggregation level. Sample metadata and group sizes used in each analysis are reported in Table S9 (guilds related) and Table S10 (beta diversity). Figure 2 (Circos) and Fig. 3 (stacked bars) summarise frequencies of occurrence derived from the plant × genus presence-absence matrix (with plot-only aggregation of low-frequency genera < 2% and < 3% for TF and LP, respectively for Fig. 3). Indicator species analysis and Raup-Crick analyses used the plant × genus presence-absence matrices. PERMANOVA, NMDS, and Mantel tests were based on Bray-Curtis dissimilarities computed from the retained plant × genus abundance matrices. Figure S2 and alpha-diversity indices were computed from the genus-level count matrix, with rarefaction based on host condition × site pooled counts.

Data wrangling and matrix preparation were carried out mainly with functions from “dplyr” [58], “tidyr” [59], “tibble” [60] and “forcats” [61] packages. The significance threshold was set at 0.05. All statistical analyses were performed using R v.4.3.1 in RStudio v. 2023.06.2 + 561.

## Results

### Fungal Genera Distribution Across Zone × Host Conditionn

To evaluate the performance of our workflow using the mock community, we compared the recovered cluster distribution across four expected genera based on equal DNA input per strain (three *Fusarium* strains and one strain each of *Botrytis*, *Alternaria* and *Aplosporella*). All genera were successfully recovered across samples, confirming the robustness of the identification pipeline at genus level. Spearman correlation coefficients between observed and expected genus-level distributions were negative (*ρ* = −0.4 to −0.7), indicating consistent rank deviations. These patterns likely reflect amplification bias, Nanopore-specific limitations, and clustering behaviour within SCATA (Table S1, Fig. S1), and they indicate that relative read abundances in field samples should be interpreted cautiously, whereas genus-level occurrence patterns are more robust. Supplementary Tables S2-S5 provide the corresponding run-level sequencing statistics, SCATA depth summaries, and island-level taxonomic assignment success underlying the curated ecological dataset.

Rarefaction curves were used as a diagnostic of sampling depth and residual genus discovery across the 85 pooled host condition × site groups in the curated genus-level dataset (Fig S2). Sequencing depth varied substantially among groups, and curves did not consistently reach an asymptote, suggesting that additional sequencing would likely recover further low-frequency genera in some groups. Observed richness ranged from 2 to 21 genera, while the number of genus-level counts contributing to each pooled group ranged from 10 to 107 (minimum thresholds: 10 counts and 2 genera). These diagnostics informed interpretation of richness-based comparisons, which were treated cautiously in light of depth heterogeneity. For example, at a pooled depth of 10 genus-level counts, low-condition plants from TF site 5 and high-condition plants from TF site 8 showed comparable genus richness (5 vs. 6 genera), illustrating that richness estimates can be very similar even at shallow pooled depth; however, we do not interpret such low-depth groups as exhaustively sampled.

Core genera were consistent across zones within and between TF and LP, with *Alternaria*, *Cladosporium*, and *Filobasidium* among the most frequently represented (Fig. 2). In TF, fungal community structure exhibited sharper zone-level segregation: coastal zones displayed strong, directional chords linking to a limited set of retained genera, suggesting potential ecological filtering or specialization. By contrast, LP presented a more balanced distribution of genus-level associations across zones, with fewer cases of single genera dominating entire zones, and more shared genera between east and west coastal sites.

The fungal assemblages in both islands were structured around a set of recurrent genera that appeared across host condition categories. Several genera such as *Alternaria*, *Aureobasidium*, *Cladosporium*, *Didymella*, *Epicoccum*, *Hormonema*, *Phaeosphaeria*, *Toxicocladosporium* and *Vishniacozyma* occurred in both high- and low-condition plants on TF and LP (11 shared genera in LP and 12 in TF; Table S12). These taxa represent a set of repeatedly detected genera associated with *C. setaceus* foliage under the conditions studied. At the same time, several genera were restricted to one host condition category within each island. Low-condition plants harboured more host condition-specific genera than high-condition plants (12 vs. 9 in LP and 13 vs. 8 in TF; Table S12). The low-condition-only sets included several genera with described plant-associated pathogens, such as *Allophoma*, *Neodevriesia*, *Nothophoma*, *Parastagonospora* or *Pseudoseptoria*, whereas many of the high-condition-only genera were yeasts (e.g. *Buckleyzyma*, *Filobasidium*, *Papiliotrema*, *Dioszegia*, *Erythrobasidium*).

Stacked bar plots provided a higher-resolution view of genus distributions across zone × host-condition groups (Fig. 3). Retained genera were defined using the site-level frequency screen described in Methods (frequency of occurrence > 1% per site, i.e. present in at least one of the six plants sampled per site), yielding 184 retained genera in TF and 126 in LP. Within each zone × host condition group, frequencies of occurrence were normalised to sum to 1. For graphical clarity, genera contributing < 2% of the normalised frequency in TF or < 3% in LP were aggregated (Fig. 3); these thresholds were applied only for visual aggregation. In TF, patterns along the coast were similar only in the southern coastal zone: low-condition plants displayed more than three times the number of low-abundance genera than their high-condition analogues (78 vs. 22). In the hill zones, low- and high-condition plants showed comparable values (hill north zone: 41 vs. 36; hill south zone: 48 vs. 49). In LP, high-condition plants in coastal zones showed no genera aggregated under < 3%. In contrast, low-condition plants in coastal zones contained a larger tail of low-frequency genera, with 23 genera aggregated on the east coast and 28 on the west. In hill zones, eastern exposures showed more low-frequency genera than the western exposures across both host condition categories (low-condition: 45 vs. 25; high-condition: 54 vs. 20, respectively).

### Fungal Indicator Genera and Guild Composition

Indicator species analysis identified 37 genera on TF and 24 on LP associated with specific zone × host condition combinations (Table S11).

On TF, coastal low-condition plants were linked to genera such as *Ramimonilia* (saprotroph; A = 0.64, B = 0.22) and *Stenella* (plant pathogen; A = 0.77, B = 0.33) (*p* < 0.05), reflecting possible colonisation under stressful conditions. In contrast, high-condition plants in the northern hills hosted diverse and specific saprotrophs and endophytes, including *Erythrobasidium* and *Xenoramularia* with high specificity (A = 0.86 and 1, respectively; *p* < 0.05), while *Symmetrospora*, *Filobasidium*, and *Vishniacozyma* were frequently detected (B = 0.91, 0.82, and 0.73, respectively; *p* < 0.05). In low-condition groups, pathogen-rich assemblages were more common in the north, while saprotrophs dominated the southern hills.

On LP, indicator genera differed between eastern and western coastal sites. Eastern low-condition plants hosted mainly saprotrophs (e.g. *Vanderaaea*, *Ramimonilia*, *Cippumomyces*), while western coasts showed a balance between saprotrophs and pathogens. High-condition coastal plants yielded few indicator taxa. In the eastern hills, both plant condition groups were associated with unique genera spanning endophytic, saprotrophic, and pathogenic guilds. Western hill sites yielded fewer indicators, with *Alternaria* (plant pathogen, B = 0.89, *p* < 0.05) notable in low-condition plants.

**Fig. 4:**
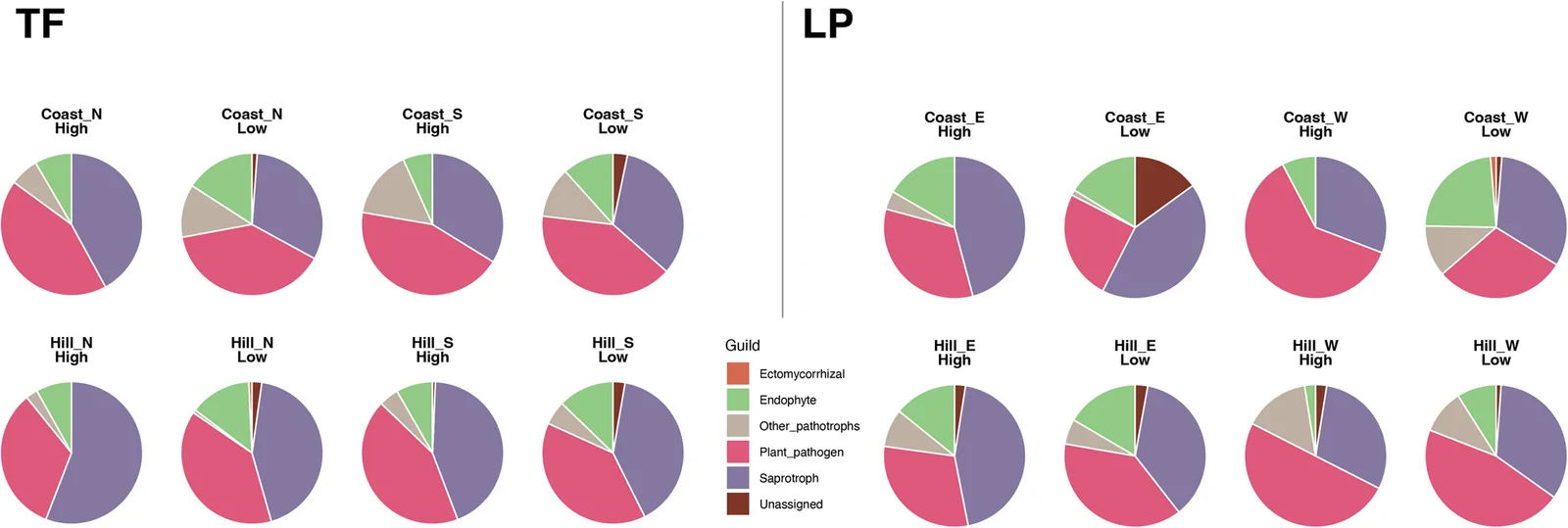
Functional guild composition of retained fungal genera in TF (left) and LP (right), shown for each zone × host-condition group. Pies were calculated from the full retained genus matrix prior to any graphical aggregation and sum to 100% within each group. Genera were assigned using FUNGuild and grouped into six guild categories: plant pathogen, other pathotrophs, saprotroph, endophyte, ectomycorrhizal and unassigned. Unassigned includes retained genera lacking assignment in the database

Guild composition analyses were based on all retained genera matched to FUNGuild. After annotation, 184 retained genera in TF and 126 in LP were evaluated for guild assignment; 12 genera per island remained unassigned (no data available in the database; Table S12). The pies represent 100% of the retained community within each zone × host-condition group, partitioned into plant pathogen, saprotroph, endophyte, other pathotroph (lichen, fungal etc.), ectomycorrhizal and unassigned categories (Fig. 4; Table S13). On TF, saprotrophs and plant pathogens dominated most groups, while endophytes were consistently subordinate and unassigned genera remained rare. The broadest representation occurred in southern coastal high-condition plants and northern hill low-condition plants, where all six categories were present. On LP, guild structure was more uneven across groups: saprotrophs dominated eastern coastal high-condition plants, whereas plant pathogens were proportionally highest in western coastal and hill groups. Endophytes remained present in all groups but reached their highest contribution in western coastal low-condition plants, while ectomycorrhizal assignments were rare (2 genera) and restricted to a small number of groups (i.e. low-condition on the western coast of LP and northern hill of TF).

### Fungal Community Diversity

Across both TF and LP, high-condition plants exhibited lower fungal richness compared to low-condition plants (Hill0: TF = 95 vs. 146; LP = 76 vs. 94; Dunn’s post-hoc test, *p* < 0.05; Table S14). However, Shannon was higher in high-condition plants in LP (3.29 vs. 2.98, Tukey’s post hoc test, *p* < 0.05), while the opposite was observed in TF (3.10 vs. 3.28, Dunn’s post hoc test, *p* < 0.05), suggesting that the drivers of diversity may differ between islands.

In TF, low-condition plants consistently harboured richer fungal communities across all zones (Dunn’s post-hoc test, *p* < 0.05; Table S14), with the highest differences observed along the coast (Hill0: north coast = 62 vs. 37; south coast = 89 vs. 36). Shannon diversity remained largely similar, except on the southern coast, where low-condition plants were significantly more diverse (3.17 vs. 2.56, Dunn’s post hoc test, *p* < 0.05).

In LP, high condition plants in the eastern hills showed substantially higher richness (Hill0 = 64 vs. 26, Dunn’s post-hoc test, *p* < 0.05) and diversity (Shannon = 3.29 vs. 2.42, Tukey’s post hoc test, *p* < 0.05) than their western counterparts (Table S13). Across the island, hill zones supported consistently more diverse fungal communities than coastal sites, with the eastern hills emerging as a recurrent hotspot. A similar hill-coast gradient appeared in TF, especially in the southern hills. While differences related to host condition were less distinct, low-condition plants on the drier western coast showed significantly higher diversity than their high-condition counterparts (Shannon = 2.78 vs. 1.61, Tukey’s post-hoc test, *p* < 0.05).

### Drivers of Fungal Community Composition: Plant Condition or Environmental Zone?

Community level differences in fungal composition reflected both host condition and environmental zone, although their relative importance varied between islands.

**Fig. 5:**
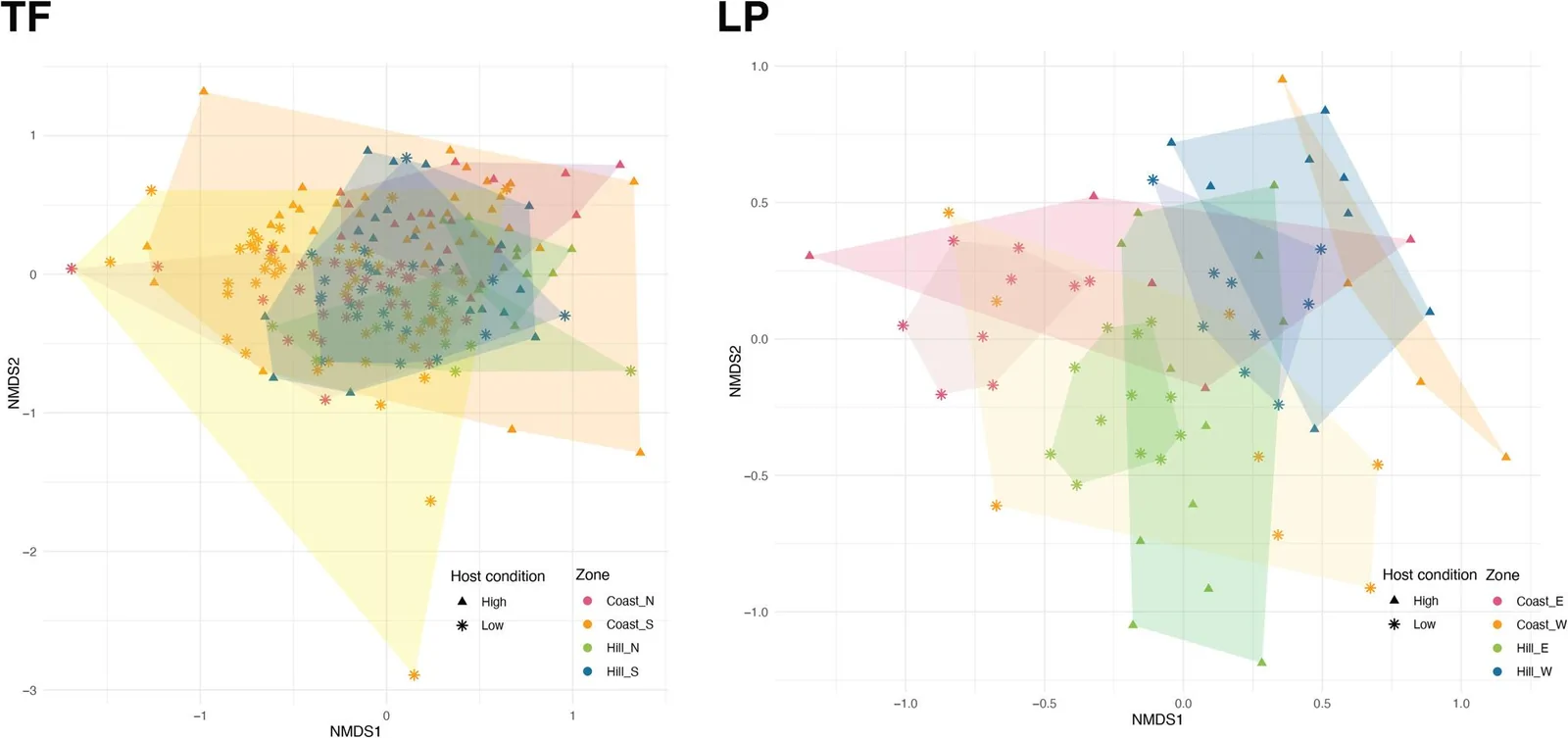
NMDS ordination of fungal communities per zone × host-condition group in TF (left) and LP (right), based on the retained genus-count matrices used for community-composition analyses. Convex hulls delineate groups with at least three replicates per host condition × zone combination

On TF, PERMANOVA showed that host condition explained the largest share of compositional variance (pseudo-F_1,196_ = 20.16, R^2^ = 0.086, *p* = 0.001), followed by zone (pseudo-F_3,196_ = 3.81, R^2^ = 0.049, *p* = 0.001) and the host condition × zone interaction (pseudo-F_3,196_ = 1.93, R^2^ = 0.025, *p* = 0.001). NMDS ordination showed moderate stress (0.194), and suggested that coastal low-condition plants, particularly in the southern coastal zone, were more clearly separated from other groups than hill samples, which showed broader overlap (Fig. 5).

On LP, both host condition and zone were also significant, but zone accounted for a slightly larger proportion of variance than host condition (zone: pseudo-F_3,61_ = 4.60, R^2^ = 0.150, *p* = 0.001; host condition: pseudo-F_1,61_ = 11.34, R^2^ = 0.123, *p* = 0.001), with a smaller yet significant interaction term (pseudo-F_3,61_ = 1.89, R^2^ = 0.062, *p* = 0.001). NMDS ordination also showed moderate stress (0.213) and indicated greater overlap among hill samples than among coastal groups, while the clearest compositional separation was observed between low- and high-condition plants on the western coast (Fig. 5).

Raup-Crick dissimilarities indicated that most pairwise comparisons fell within the intermediate range, consistent with a substantial stochastic component in fungal community assembly. On Tenerife, 18.681 of 20.706 unique sample pairs were classified as stochastic, compared with 1869 heterogeneous and 156 homogenous pairs; the distribution was centred in the intermediate-positive range (median = 0.193, mean = 0.180). On La Palma, 2034 of 2346 unique pairs were stochastic, with 260 heterogeneous and 52 homogeneous pairs, but the distribution was shifted toward higher positive values (median = 0.495, mean = 0.314), suggesting relatively stronger turnover among sample pairs than on Tenerife. Variation partitioning showed that both host condition and environmental zone contributed significantly to community composition, but their relative importance differed between islands. On Tenerife, the pure fraction explained by host condition (adj. R^2^= 0.0685, *p* = 0.001) exceeded that explained by zone (adj. R^2^ = 0.0299, *p* = 0.001) and the full model accounted for 9.9% of adjusted variance. On La Palma, host condition (adj. R^2^ = 0.1132, *p* = 0.001) and zone (adj. R^2^ = 0.1029, *p* = 0.001) contributed similarly, and the full model explained 20.7% of adjusted variance.

Mantel tests based on Bray-Curtis dissimilarities from the retained genus-count matrices further supported island-specific differences in the correlates of fungal composition. On Tenerife, abundance-based fungal dissimilarity correlated most strongly with host condition (*r* = 0.3089, *p* = 0.001), while zone alone was not significant (*r* = 0.0207, *p* = 0.104). The combined condition × zone grouping was also significant, but weaker than condition alone (*r* = 0.1539, *p* = 0.001). That pattern is coherent with the PERMANOVA and VPA results, where host condition explained more variance than zone on TF. On La Palma, all three Mantel tests were significant, with the strongest association again for host condition (*r* = 0.3805, *p* = 0.001), followed by the combined grouping (*r* = 0.3592, *p* = 0.001) and zone (*r* = 0.2455, *p* = 0.001). So, LP is not a case where condition disappears and only environment remains; rather, both factors are associated with community dissimilarity, with host condition still slightly stronger in this Mantel formulation.

Geographic distance was also significantly associated with community dissimilarity on both islands, although the correlations were modest (TF: *r* = 0.1795, *p* = 0.001; LP: *r* = 0.1464, *p* = 0.001; 999 permutations), indicating that fungal communities became more dissimilar with increasing spatial separation.

Across both islands, fungal communities in coastal zones were more sensitive to plant condition, with clear compositional shifts between low- and high-condition groups. In contrast, hill sites—particularly in humid or buffered exposures—exhibited greater compositional similarity across host condition levels, suggesting increased community stability.

### Plant Condition and Zone Effects on Guild Richness

Generalized linear mixed models showed that fungal guild richness was shaped by both plant condition and environmental zone, although the strength and form of these effects differed between islands (Table S15).

In TF, all retained models were additive. Saprotroph richness was best described by a negative-binomial GLMM including plant condition and zone (AIC = 868.4). Although the interaction model had a slightly lower AIC (867.19), its additional complexity was not supported (χ² = 7.22, df = 3, *p* < 0.065), so the additive model was retained. Within this model, low condition plants hosted more saprotrophic genera than high-condition hosts (Estimate = 0.419, *z* = 3.57, *p* = 0.001), corresponding to an estimated 52% increase in expected richness, relative to the northern coast baseline. Saprotroph richness was also higher in northern hill sites (Estimate = 0.815, *z* = 2.12, *p* = 0.034), whereas southern coastal sites showed a marginal decrease (Estimate = −0.480, *z* = −1.71, *p* = 0.087). Endophyte richness on TF was also best described by an additive Poisson GLMM (AIC = 465.7), while the interaction model was not supported (χ² = 1.40, df = 3, *p* = 0.705). Low-condition plants hosted substantially more endophytic genera than high-condition plants (Estimate = 1.03, *z* = 5.81, *p* < 0.001), corresponding to an estimated 2.8-fold increase in expected richness. Endophyte richness was lower on the southern coast than on the northern coast (Estimate = −0.54, *z* = −2.25, *p* = 0.024), whereas hill zones showed no significant effect. Plant pathogen richness on TF was likewise best described by an additive Poisson GLMM (AIC = 822.8), and the interaction model was not retained (χ² = 3.34, df = 3, *p* = 0.342). Low-condition plants hosted more pathogen genera than high-condition plants (Estimate = 0.46, *z* = 5.48, *p* < 0.001), corresponding to an estimated 59% increase in expected richness. Zone effects were weak overall: southern coastal sites showed only a marginal reduction relative to the northern coast (Estimate = −0.24, *z* = −1.70, *p* = 0.089), while hill zones did not differ significantly from the baseline.

On LP, the responses were more guild dependent. Saprotroph richness was best explained by a negative binomial GLMM including the interaction between plant condition and zone (AIC = 318.3), which improved fit relative to the additive model (χ² = 9.20, df = 3, *p* = 0.027). Relative to the baseline group of high-condition plants from the eastern coast, saprotroph richness was higher in high-condition plants from eastern hills (Estimate = 0.98, *z* = 2.11, *p* = 0.035), corresponding to an estimated 2.66-fold increase. In contrast, high-condition plants from the western coast showed a marginal reduction in richness (Estimate = −1.18, *z* = −1.7, *p* = 0.09), equivalent to an estimated 70% decrease. Endophyte richness on LP also followed an interaction model (AIC = 188), which improved fit relative to the additive model (χ² = 10.64, df = 3, *p* < 0.014). However, individual coefficients within the retained model did not reach conventional significance thresholds. The strongest trends suggested lower endophyte richness in low-condition plants from the western coast through the interaction term (Estimate = 2.04, *z* = 1.74, *p* = 0.082), indicating a context-dependent response of endophyte richness across zones. By contrast, plant-pathogen richness on LP was best described by an additive Poisson GLMM (AIC = 277.8), and the interaction model was not supported (ΔAIC = 4.5, *p* = 0.681). Within the retained model, low-condition plants hosted 56% more pathogen genera than high-condition plants (Estimate = 0.44, *z* = 3.25, *p* = 0.001). Pathogen richness was also higher in hill zones, with an estimated 2.6-fold increase in the eastern hills (Estimate = 0.95, *z* = 4.22, *p* < 0.01) and a 1.9-fold increase in the western hills (Estimate = 0.64, *z* = 2.62, *p* = 0.009) relative to the eastern coast baseline.

Overall, these models support a strong association between low plant condition and increased guild richness on TF, where all three focal guilds responded positively to host deterioration. On LP, plant pathogens also increased in low-condition plants, whereas saprotrophs and endophytes showed a stronger zone dependence, suggesting that environmental context modulated host-fungus associations in a guild specific manner.

## Discussion

A limitation of this study is that the environmental stratification used to approximate humid versus drier conditions, based on orientation relative to prevailing winds and altitude, is spatially structured and therefore potentially pseudoreplicated. Sampling sites within a zone cannot be treated as fully independent replicates of a “humid” or “dry” treatment, and the number of sites was also unbalanced across zones within each island.

For this reason, zone-related patterns are interpreted as broad, island-specific associations between fungal community structure and environmental context rather than as definitive causal contrasts between independently replicated humidity regimes. Even so, plant level replication within sites, together with coverage across the principal environmental contexts on each island, allows a consistent view of how host condition and environment relate to phyllosphere fungal assembly. Taken together, our results indicate that both factors contributed to fungal community assembly, but not in an equivalent manner across islands. On Tenerife, host condition was more strongly related to community composition and guild richness, whereas on La Palma the environmental gradient was more clearly expressed, particularly in buffered eastern hill zones. At the same time, Raup-Crick patterns on both islands remained largely within the stochastic range, indicating that deterministic filtering did not replace substantial turnover among samples. Rather than supporting a single dominant mechanism, the dataset points to context-dependent filtering against a broadly stochastic background. Comparable dynamics have also been reported in another *Cenchrus* species, *C. ciliaris*, in which foliar fungal communities reflected opportunistic acquisition from the environment together with host and environmental filtering [62]. Within this framework, the higher richness often observed in low-condition plants suggests that physiologically compromised hosts may be less selective toward incoming fungi, allowing broader colonisation by saprotrophs, endophytes and, in some cases, pathogens. This pattern was most evident in exposed coastal settings, where host stress and environmental harshness likely acted together. Sun et al. [63] found that phyllosphere fungal diversity increases with cuticular leakage, highlighting how structural traits can affect microbial niche availability. Marčiulynas [64] reported reduced fungal diversity in diseased *Ulmus glabra* trees affected by Dutch elm disease. In our case, the absence of visible disease suggests that compromised hosts may tolerate or facilitate broader fungal colonisation, possibly through weakened defences, nutrient leakage, or altered surface chemistry.

By contrast, more buffered hill environments, particularly in northern Tenerife and eastern La Palma, appeared either to reduce the contrast between host-condition classes or to enhance richness independently of host condition. In this sense, host deterioration and environmental buffering did not produce the same outcome: the former was associated mainly with broader and less predictable colonisation, whereas the latter was associated more with greater opportunity for persistence and guild enrichment. This was especially evident on LP, where environmental effects were more prominent than host condition and eastern hill sites consistently supported more diverse communities, especially among high-condition plants. This agrees with Dea et al. [14], who showed that humidity and precipitation can increase fungal richness regardless of host identity. Conversely, lower diversity in high-condition plants under dry conditions may reflect selective microbial partnerships, possibly favouring functional specialisation [12].

Across both islands, a stable core fungal community included genera such as *Alternaria*, *Cladosporium*, and *Filobasidium*, which were frequently detected across zones and host condition categories. These fungi are ecologically versatile and may function as epiphytes, endophytes, or saprotrophs depending on context [65], which may help explain their persistence under contrasting host and environmental conditions.

Similar patterns have been reported in other heterogeneous landscapes, such as north-west Italy [66]. Against this shared background, indicator genera and guild patterns suggest that host condition influenced not only how many fungi were present, but also what kinds of associations became established. On TF, low-condition coastal plants were linked to genera such as *Stenella*, pointing to assemblages favoured under exposed or physiologically stressed conditions. Cercosporoid fungi are broadly treated as important plant pathogens [67], and *Stenella* is explicitly discussed with taxa from foliar disease contexts [68–70]. By contrast, high-condition plants in the northern hills were associated with more specific yeast-like and recurrent phyllosphere-associated taxa, including *Symmetrospora* [71], *Filobasidium* [72–74] and *Erythrobasidium* [75]. Although not restricted to a single group, *Vishniacozyma* was also frequent in high-condition plants and has been associated with antagonistic or endophytic roles in cool environments [64]. Together, these patterns suggest that, on TF, better host condition in buffered environments may favour more recurrent fungal associations, whereas stressed plants, particularly in exposed coastal zones, may allow broader colonisation by fungi with more opportunistic or context-dependent lifestyles. A similar contrast emerged on LP, but with a stronger environmental component. Eastern coastal low-condition plants were associated mainly with saprotrophs, whereas western coastal sites showed a more mixed signal that included stress-related pathogenic taxa. In the eastern hills, both host-condition groups were linked to distinctive genera spanning saprotrophic, endophytic and pathogenic guilds, while western hill groups yielded fewer indicators overall. This east-west asymmetry is consistent with the broader signal observed on LP, where environmentally buffered zones appeared to structure fungal occurrence more strongly than host condition alone. In this sense, LP did not simply mirror TF; rather it showed that recurrent fungal associations may emerge through different pathways depending on how host condition and local environment combine. Although the present design does not allow island-level causes to be separated formally, the contrasting patterns observed on TF and LP may partly reflect differences in how trade-wind exposure, topography and local habits mosaics are arranged on each island. Such differences could influence whether host condition or environmental buffering becomes the more visible correlate of fungal assembly in a given island context.

Guild composition and guild-richness models reinforced this interpretation. On TF, low-condition plants hosted significantly higher richness of saprotrophs, endophytes and pathogens, indicating that host deterioration was associated with broader functional representation. In contrast, buffered hill zones, particularly in the north, contributed mainly to saprotroph and endophyte richness, suggesting that favourable microclimatic conditions can modulate, but not fully override, host-related filtering. On LP, guild richness was more context-dependent: saprotrophs and endophytes responded to host condition differently across zones, while pathogen richness increased both in low-condition plants and in hill environments, especially in the east. This agrees with the view that fungal guilds are not fixed ecological compartments, but may shift or overlap depending on host condition and environmental setting [17].

For an invasive grass such as *Cenchrus setaceus*, these results suggest that fungal communities linked to poor versus good host condition are not interchangeable. Low-condition plants tended to host richer and often less specific guild assemblages, including higher representation of pathogens and saprotrophs, whereas high-condition plants more often retained recurrent associations in buffered zones. This distinction may be relevant when searching for naturally associated fungi of possible management interest. The present data do not identify candidate biocontrol agents directly, but they do indicate that low-condition hosts, particularly in exposed coastal settings, may provide a useful ecological window for detecting fungi associated with host weakening, while buffered hill environments may be more informative for understanding which taxa persist under relatively stable host-fungus relationships. In that sense, the value of these fungal patterns lies not only in describing community assembly, but also in helping to frame where naturally associated taxa of future management interest are more likely to be detected [13, 76].

## Conclusions

Fungal community assembly in *Cenchrus setaceus* reflected a predominantly stochastic background on both islands, but with consistent non-random structure associated with host condition and environmental context. On Tenerife, host condition was more strongly related to community composition and guild richness, whereas on La Palma the environmental gradient, particularly in buffered eastern hill zones, was more clearly expressed. Low-condition plants generally hosted richer and more variable fungal assemblages, while buffered environments were more often associated with guild enrichment and more recurrent fungal associations.

Together, these results suggest that phyllosphere communities on this invasive grass reflect context-dependent filtering against a background of substantial turnover, rather than a single dominant assembly process. This pattern helps clarify how host state and local environment jointly influence fungal community structure and may also help guide future searches for naturally associated fungi linked either to host weakening or to more persistent host-fungus relationships.

## Supplementary Information

Below is the link to the electronic supplementary material.

Supplementary Material 1 (DOCX 245 KB)

## Data Availability

The raw sequence reads generated during the current study have been deposited in the NCBI Sequence Read Archive under BioProject accession number PRJNA1294559. The processed data supporting the analyses are provided in the article and its Supplementary Information. Additional analysis files are available from the corresponding author upon reasonable request.

## Declarations

### Appropriate Permissions and/or Licences

*C. setaceus* plant material was handled under the authorisation to access Spanish plant genetic resources from wild taxa issued by Ministerio para la Transición Ecológica y Reto Demográfico, and collected under the authorisations no. EEI-006/2019 (no. 1959553) and no. ESNC54, issued by the Consejería de Transición Ecológica, Lucha contra el Cambio Climático y Planificación Territorial of the Gobierno de Canarias. The collection and handling of plant material complied with the Nagoya Protocol and all relevant regulations.
