# Supplementary material for: Shaping Fungal Communities in Cenchrus setaceus: Host Condition and Habitat Filtering
Source: Microb Ecol. 2026 Jun 16;89:163. doi: 10.1007/s00248-026-02805-3 (PMC13500446; doi:10.1007/s00248-026-02805-3)
Supplement: Supplementary file 1 [file 248_2026_2805_MOESM1_ESM.docx]

Supplementary Material

Shaping fungal communities in *Cenchrus setaceus*: host condition and habitat filtering

**Andreea Cosoveanu^1,2^, Mario A. González-Carracedo^3^, Jorge Sopena Lasala^1^, José A. Pérez Pérez^3^, Raimundo Cabrera^1*^**

^1^ Department of Botany, Ecology and Plant Physiology, Faculty of Sciences, Section Biology, Universidad de La Laguna, 38206 La Laguna, Tenerife, Spain.

^2^ Gabinete de Estudios Ambientales (GEA), S.L.U, 38659, Tenerife, Canary Islands, Spain.

^3^ Department of Biochemistry, Microbiology, Cellular Biology and Genetics, Faculty of Sciences, Section Biology, Universidad de La Laguna, 38206 La Laguna, Tenerife, Spain.

* Correspondence: rcabrera@ull.edu.esacosovea@ull.edu.es

Original Article submitted to Microbial Ecology

| Table S1 Cluster counts based on the SCATA cluster representatives and subsequent BLAST identification per genus per mock sample (n = 7). The Spearman correlation coefficient (ρ) compares the observed genus rank distribution with the expected uniform rank (equal input across four genera). Values: ~ 1 – perfect agreement with expected ranks; ~ 0 – no rank correlation; negative values reflect deviation in recovery across genera | | | | | | |
| --- | --- | --- | --- | --- | --- | --- |
| Sample | *Fusarium* | *Botrytis* | *Aplosporella* | *Alternaria* | Other | Spearman (*p*) |
| 1 | 14 | 11 | 1 | 13 | 0 | -0.4 |
| 2 | 45 | 46 | 12 | 42 | 0 | -0.6 |
| 3 | 20 | 12 | 3 | 12 | 0 | -0.6 |
| 4 | 55 | 30 | 14 | 44 | 1 | -0.4 |
| 5 | 22 | 11 | 7 | 16 | 1 | -0.4 |
| 6 | 2 | 4 | 1 | 1 | 0 | -0.7 |
| 7 | 12 | 13 | 4 | 11 | 0 | -0.6 |

**Fig. S1** Observed rank of each genus per mock sample based on the number of SCATA cluster representatives. A lower rank (closer to 1) indicates higher relative representation. The red dashed line (2.5) indicates the expected rank under equal DNA input across the four genera (ideal recovery). Deviation from this line reflects recovery biases in either amplification or taxonomic (above = underrepresented, below = overrepresented)

| Table S2 Run-level sequencing statistics for demultiplexed reads assigned to barcodes per plate | | | | |
| --- | --- | --- | --- | --- |
| Metric | Plate 1 | Plate 2 | Plate 3 | Plate 4 |
| Reads assigned to barcodes (number of reads) | 2,792,524.00 | 2,597,667.00 | 2,872,175.00 | 2,853,086.00 |
| Bases (bp) | 1,756,183,357.00 | 1,707,148,474.00 | 1,743,122,991.00 | 1,707,003,760.00 |
| Bases (Gb) | 1.76 | 1.71 | 1.74 | 1.71 |
| Mean read length (bp; bases/reads) | 628.90 | 657.20 | 606.90 | 598.30 |
| Standard deviation of read length (bp) | 141.90 | 118.80 | 58.30 | 57.70 |
| N50 (bp) | 690.00 | 695.00 | 589.00 | 587.00 |
| Mean Q | 11.40 | 11.58 | 11.70 | 11.60 |
| Reads >Q7 | 2,791,056 (99.9%) | 2,596,782 (100%) | 2,872,098 (100.0%) | 2,853,020 (100.0%) |
| Reads >Q10 | 2,309,576 (82.7%) | 2,205,571 (84.91%) | 2,460,397 (85.7%) | 2,432,966 (85.3%) |
| Reads >Q12 | 930,511 (33.3%) | 1,013,731 (39.02%) | 1,293,641 (45.0%) | 1,110,530 (38.9%) |
| Reads >Q15 | 37,094 (1.3%) | 32,854 (1.26%) | 37,370 (1.3%) | 17,601 (0.6%) |

* Run-level sequencing metrics for reads assigned to barcodes (i.e. reads that NanoPlot/NanoStats detected inside barcode bins; unclassified reads are not included). For each plate, the table reports total demultiplexed reads (number of reads), total bases, mean read length, standard deviation and N50 of read length, mean quality score and the number and percentage of reads with Q scores above 7, 10, 12 and 15, as reported by Nanostats.

| Table S3 Per-sample read depth before and after SCATA quality control across MinION plates (n = 379 barcodes/samples) | | | | | | | | |
| --- | --- | --- | --- | --- | --- | --- | --- | --- |
| Plate ID | Demultiplexed barcode-assigned reads per sample (min) | Demultiplexed barcode-assigned reads per sample (max) | Demultiplexed barcode-assigned reads per sample (median) | Reads passing QC per sample (min) | Reads passing QC per sample (max) | Reads passing QC per sample (median) | OTU clusters produced by SCATA |  |
| 1 | 649 | 161451 | 24762.5 | 7 | 105522 | 11527.5 | 3572 |  |
| 2 | 270 | 122851 | 23177.5 | 2 | 53986 | 10297.5 | 3199 |  |
| 3 | 143 | 79859 | 28637 | 1 | 47616 | 16492 | 4688 |  |
| 4 | 102 | 75186 | 31155 | 2 | 50658 | 19757 | 4999 |  |

* For each plate, values represent the distribution across samples (barcodes) within that plate: minimum, median, and maximum numbers of demultiplexed barcode-assigned reads, and minimum, median and maximum numbers of reads passing SCATA quality control. “OTU clusters produced by SCATA” refers to the number of OUT clusters obtained from SCATA clustering for that plate.

| Table S4 Taxonomic assignment success by island for SCATA-provided representative sequences retained in the curated ecological workflow after BLAST curation and Vu et al threshold filtering | | | | | | | | | |
| --- | --- | --- | --- | --- | --- | --- | --- | --- | --- |
| Island | SCATA representative sequences provided | Assigned at family | Assigned at genus | Unassigned at genus | Assigned at species | % assigned at family | % assigned at genus | % assigned at species | Unassigned at genus |
| Control | 8 | 6 | 6 | 2 | 0 | 75 | 75 | 0 | 25 |
| LP | 3215 | 3106 | 1946 | 1269 | 24 | 96.61 | 60.53 | 0.75 | 39.47 |
| TF | 7452 | 7225 | 4280 | 3172 | 70 | 96.95 | 57.43 | 0.94 | 42.57 |
| * Counts are based on SCATA representative sequences linked to the curated genus-level dataset used for downstream ecological analyses, not on all clusters produced by SCATA. | | | | | | | | | |

| Table S5 SCATA yield by island for barcode runs retained in the curated ecological workflow: quality-control summary, read-depth distribution, cluster yield and read-length statistics | | | | | | | | | | | | | | | | | | |
| --- | --- | --- | --- | --- | --- | --- | --- | --- | --- | --- | --- | --- | --- | --- | --- | --- | --- | --- |
| Island | No. of barcodes | Total pyro reads | No. of seq pass QC / % | No. of reads discarded / % | Reads too short | Total pyro reads (min) | Total pyro reads (median) | Total pyro reads (max) | Seq pass QC (min) | Seq pass QC (median) | Seq pass QC (max) | Global clusters | No. of ident clusters / % | Global singletons | Ident singletons | Mean readlength (mean) | Mean readlength (min) | Mean readlength (max) |
| Control | 4 | 8259 | 1868 / 22.62% | 6391 / 77.38% | 1143 | 788 | 1913.5 | 3644 | 101 | 434 | 899 | 9 | 1 / 11.11% | 1825 | 2 / 0.11% | 427.50 | 382 | 461 |
| LP | 68 | 2163535 | 1284425 / 59.37% | 879110 / 40.63% | 506203 | 5616 | 30938.5 | 70368 | 3361 | 17471 | 48300 | 3747 | 126 / 3.36% | 1241809 | 438 / 0.04% | 424.47 | 386 | 520 |
| TF | 203 | 6487434 | 3456927 / 53.29% | 3030507 / 46.71% | 1687571 | 549 | 28301 | 122851 | 248 | 15406 | 53986 | 9377 | 324 / 3.46% | 3330160 | 1024 / 0.03% | 421.00 | 364 | 469 |
|  | * Values summarise only barcode runs linked to the curated dataset used for downstream ecological analyses. Full per-plate depth summaries across all 379 barcode-assigned samples are reported in Table S3. | | | | | | | | | | | | | | | | | |

| Table S6 Sampling-site footprint and within-site plant spacing (UTM-based) | | | | | | | | | | |  |
| --- | --- | --- | --- | --- | --- | --- | --- | --- | --- | --- | --- |
| Island | Site_ID | Zone | Position | Cardinal | Nr. of plants | Altitude  (m a.s.l) | Sampling date | Site area (m2) | Maximum inter-plant distance (m) | Median nearest-neighbour distance (m) | |
| LP | MLP1 | Hill_E | Hill | E | 6 | 527 (521-529) | 2022-05-31 | 290 | 52.26 | 12.17 | |
| LP | MLP10 | Coast_E | Coast | E | 6 | 153 (153-153) | 2022-06-06 | 135 | 55.01 | 6.08 | |
| LP | MLP11 | Hill_E | Hill | E | 6 | 365 (359-366) | 2022-06-06 | 91 | 57.58 | 7.35 | |
| LP | MLP12 | Coast_W | Coast | W | 6 | 292 (281-294) | 2022-06-07 | 642 | 93.71 | 11.86 | |
| LP | MLP13 | Coast_W | Coast | W | 6 | 156.5 (150-159) | 2022-06-07 | 488 | 43.07 | 9.68 | |
| LP | MLP2 | Hill_E | Hill | E | 6 | 458.5 (456-460) | 2022-05-31 | 70 | 41.16 | 2.92 | |
| LP | MLP3 | Coast_E | Coast | E | 6 | 35.5 (33-38) | 2022-05-31 | 736 | 56.18 | 7.63 | |
| LP | MLP4 | Coast_E | Coast | E | 6 | 228.5 (227-249) | 2022-05-31 | 639 | 120.15 | 2.59 | |
| LP | MLP5 | Hill_W | Hill | W | 6 | 342.5 (338-353) | 2022-06-01 | 496 | 80.92 | 5.68 | |
| LP | MLP6 | Hill_W | Hill | W | 6 | 350 (340-357) | 2022-06-01 | 1419 | 97.02 | 12.72 | |
| LP | MLP7 | Hill_W | Hill | W | 6 | 679 (675-683) | 2022-06-01 | 1369 | 77.82 | 14.15 | |
| LP | MLP8 | Coast_W | Coast | W | 6 | 163 (163-163) | 2022-06-06 | 424 | 70.54 | 11.09 | |
| LP | MLP9 | Hill_E | Hill | E | 6 | 508 (508-508) | 2022-06-06 | 73 | 38.08 | 1.41 | |
| TF | MTF12 | Coast_S | Coast | S | 6 | 126 (126-126) | 2022-05-02 | 250 | 35.13 | 7.07 | |
| TF | MTF13 | Hill_S | Hill | S | 6 | 586 (586-586) | 2022-05-05 | 228 | 51.13 | 9.00 | |
| TF | MTF15 | Coast_S | Coast | S | 6 | 28 (28-28) | 2022-05-05 | 305 | 31.31 | 5.83 | |
| TF | MTF16 | Hill_S | Hill | S | 6 | 377 (377-377) | 2022-05-02 | 24 | 9.50 | 2.24 | |
| TF | MTF18 | Coast_S | Coast | S | 6 | 26 (26-26) | 2022-05-02 | 258 | 38.45 | 9.50 | |
| TF | MTF19 | Hill_S | Hill | S | 6 | 581 (581-581) | 2022-05-03 | 100 | 25.71 | 4.12 | |
| TF | MTF20 | Coast_S | Coast | S | 6 | 6 (6-6) | 2022-05-03 | 563 | 43.57 | 13.68 | |
| TF | MTF21 | Coast_S | Coast | S | 6 | 291 (291-291) | 2022-05-03 | 281 | 24.84 | 7.07 | |
| TF | MTF23 | Hill_S | Hill | S | 6 | 348 (348-348) | 2022-05-03 | 86 | 13.60 | 4.35 | |
| TF | MTF24 | Coast_S | Coast | S | 6 | 47 (47-47) | 2022-05-03 | 81 | 53.01 | 8.49 | |
| TF | MTF28 | Hill_S | Hill | S | 6 | 525 (525-525) | 2022-05-09 | 94 | 32.08 | 3.53 | |
| TF | MTF29 | Coast_S | Coast | S | 6 | 20 (20-20) | 2022-05-09 | 550 | 60.74 | 5.71 | |
| TF | MTF32 | Coast_S | Coast | S | 6 | 75 (75-75) | 2022-05-09 | 2216 | 127.43 | 14.76 | |
| TF | MTF34 | Coast_S | Coast | S | 6 | 5 (5-5) | 2022-05-09 | 37 | 21.83 | 5.03 | |
| TF | MTF37 | Coast_S | Coast | S | 6 | 5 (5-5) | 2022-05-10 | 1110 | 156.52 | 13.73 | |
| TF | MTF39 | Coast_S | Coast | S | 6 | 300 (300-300) | 2022-05-10 | 37 | 15.50 | 2.68 | |
| TF | MTF4 | Coast_S | Coast | S | 6 | 33 (33-33) | 2022-04-25 | 82 | 24.45 | 3.48 | |
| TF | MTF40 | Coast_S | Coast | S | 6 | 15 (15-15) | 2022-05-10 | 27 | 10.04 | 3.22 | |
| TF | MTF44 | Hill_S | Hill | S | 6 | 660 (660-660) | 2022-05-10 | 9 | 8.87 | 1.52 | |
| TF | MTF46 | Coast_S | Coast | S | 6 | 14 (12-16) | 2022-05-24 | 300 | 26.01 | 9.19 | |
| TF | MTF48 | Hill_S | Hill | S | 6 | 811 (808-815) | 2022-05-24 | 2177 | 158.89 | 15.62 | |
| TF | MTF5 | Coast_S | Coast | S | 6 | 219 (219-219) | 2022-04-25 | 22 | 17.12 | 4.19 | |
| TF | MTF51 | Coast_N | Coast | N | 6 | 160.5 (147-165) | 2022-05-23 | 399 | 40.55 | 1.07 | |
| TF | MTF52 | Coast_N | Coast | N | 6 | 55 (51-60) | 2022-05-23 | 1185 | 67.59 | 16.55 | |
| TF | MTF56 | Coast_N | Coast | N | 6 | 10.5 (10-18) | 2022-05-17 | 267 | 39.57 | 5.41 | |
| TF | MTF57 | Coast_N | Coast | N | 6 | 183 (180-188) | 2022-05-17 | 263 | 74.25 | 10.20 | |
| TF | MTF59 | Coast_N | Coast | N | 6 | 183 (181-188) | 2022-05-17 | 2976 | 152.88 | 8.22 | |
| TF | MTF6 | Coast_S | Coast | S | 6 | 37 (37-37) | 2022-04-25 | 57 | 12.81 | 3.86 | |
| TF | MTF64 | Hill_N | Hill | N | 6 | 369.5 (369-371) | 2022-05-16 | 184 | 50.70 | 6.91 | |
| TF | MTF65 | Coast_N | Coast | N | 6 | 22 (19-24) | 2022-05-16 | 454 | 52.26 | 9.59 | |
| TF | MTF68 | Coast_N | Coast | N | 6 | 182 (175-188) | 2022-05-17 | 602 | 98.88 | 7.43 | |
| TF | MTF69 | Coast_N | Coast | N | 6 | 278 (273-280) | 2022-05-16 | 191 | 34.72 | 4.17 | |
| TF | MTF7 | Coast_S | Coast | S | 6 | 163 (163-163) | 2022-04-25 | 13 | 7.28 | 2.21 | |
| TF | MTF72 | Hill_N | Hill | N | 6 | 298.5 (296-301) | 2022-05-16 | 485 | 83.57 | 3.55 | |
| TF | MTF74 | Hill_N | Hill | N | 6 | 541.5 (537-546) | 2022-05-12 | 438 | 60.92 | 10.59 | |
| TF | MTF75 | Coast_N | Coast | N | 6 | 36 (33-43) | 2022-05-12 | 1322 | 72.93 | 9.75 | |
| TF | MTF77 | Hill_N | Hill | N | 6 | 508.5 (500-518) | 2022-05-12 | 818 | 99.34 | 12.36 | |
| TF | MTF8 | Coast_S | Coast | S | 6 | 19 (19-19) | 2022-04-25 | 86 | 18.41 | 5.74 | |
| TF | MTF9 | Hill_S | Hill | S | 6 | 534 (534-534) | 2022-04-25 | 177 | 28.83 | 7.67 | |

* Altitude is reported as median (min-max) across the six sampled plants per site. Site area is convex-hull area from UTM coordinates.

| Table S7 Plant metadata and grouping variables | | | | | | | | | | |
| --- | --- | --- | --- | --- | --- | --- | --- | --- | --- | --- |
| Sampling date | Island | Site ID | Plant ID | Host condition | Zone (exposure x altitude) | Altitude | UTM28R East (m) | UTM28R North(m) | MinION plate | MinION barcode |
| 2022-06-06 | LP | MLP10 | MLP10E1 | Low | Coast_E | 153 | 229202 | 3164357 | 4 | 83 |
| 2022-06-06 | LP | MLP10 | MLP10E2 | Low | Coast_E | 153 | 229194 | 3164360 | 4 | 84 |
| 2022-06-06 | LP | MLP10 | MLP10E3 | Low | Coast_E | 153 | 229203 | 3164351 | 4 | 85 |
| 2022-06-06 | LP | MLP10 | MLP10S1 | High | Coast_E | 153 | 229243 | 3164335 | na | na |
| 2022-06-06 | LP | MLP10 | MLP10S2 | High | Coast_E | 153 | 229210 | 3164349 | na | na |
| 2022-06-06 | LP | MLP10 | MLP10S3 | High | Coast_E | 153 | 229211 | 3164350 | 2 | 86 |
| 2022-06-06 | LP | MLP11 | MLP11E1 | Low | Hill_E | 364 | 228545.00 | 3174920.00 | 4 | 86 |
| 2022-06-06 | LP | MLP11 | MLP11E2 | Low | Hill_E | 361 | 228571.49 | 3174900.52 | 4 | 87 |
| 2022-06-06 | LP | MLP11 | MLP11E3 | Low | Hill_E | 359 | 228580.14 | 3174895.38 | 4 | 88 |
| 2022-06-06 | LP | MLP11 | MLP11S1 | High | Hill_E | 366 | 228530.96 | 3174925.33 | 2 | 87 |
| 2022-06-06 | LP | MLP11 | MLP11S2 | High | Hill_E | 366 | 228535.00 | 3174923.00 | 2 | 88 |
| 2022-06-06 | LP | MLP11 | MLP11S3 | High | Hill_E | 366 | 228534.71 | 3174922.59 | 2 | 89 |
| 2022-06-07 | LP | MLP12 | MLP12E1 | Low | Coast_W | 281 | 213708.67 | 3173062.51 | 4 | 89 |
| 2022-06-07 | LP | MLP12 | MLP12E2 | Low | Coast_W | 282 | 213710.72 | 3173061.59 | 4 | 90 |
| 2022-06-07 | LP | MLP12 | MLP12E3 | Low | Coast_W | 294 | 213747.58 | 3173099.39 | 4 | 91 |
| 2022-06-07 | LP | MLP12 | MLP12S1 | High | Coast_W | 293 | 213755.17 | 3173130.02 | 2 | 91 |
| 2022-06-07 | LP | MLP12 | MLP12S2 | High | Coast_W | 294 | 213763.35 | 3173138.61 | 2 | 92 |
| 2022-06-07 | LP | MLP12 | MLP12S3 | High | Coast_W | 291 | 213741.82 | 3173112.26 | na | na |
| 2022-06-07 | LP | MLP13 | MLP13E1 | Low | Coast_W | 157 | 213352.72 | 3173997.25 | 4 | 92 |
| 2022-06-07 | LP | MLP13 | MLP13E2 | Low | Coast_W | 156 | 213366.99 | 3174023.01 | na | na |
| 2022-06-07 | LP | MLP13 | MLP13E3 | Low | Coast_W | 158 | 213376.49 | 3174024.86 | 4 | 94 |
| 2022-06-07 | LP | MLP13 | MLP13S1 | High | Coast_W | 159 | 213362.73 | 3174002.31 | 2 | 94 |
| 2022-06-07 | LP | MLP13 | MLP13S2 | High | Coast_W | 152 | 213341.08 | 3174000.34 | 2 | 95 |
| 2022-06-07 | LP | MLP13 | MLP13S3 | High | Coast_W | 150 | 213339.51 | 3174008.90 | na | na |
| 2022-05-31 | LP | MLP1 | MLP1E1 | Low | Hill_E | 529 | 222720.84 | 3155209.97 | 4 | 58 |
| 2022-05-31 | LP | MLP1 | MLP1E2 | Low | Hill_E | 529 | 222720.55 | 3155210.01 | 4 | 59 |
| 2022-05-31 | LP | MLP1 | MLP1E3 | Low | Hill_E | 529 | 222720.84 | 3155209.97 | 4 | 60 |
| 2022-05-31 | LP | MLP1 | MLP1S1 | High | Hill_E | 525 | 222724.37 | 3155198.32 | 2 | 58 |
| 2022-05-31 | LP | MLP1 | MLP1S2 | High | Hill_E | 521 | 222715.0 | 3155172.03 | 2 | 59 |
| 2022-05-31 | LP | MLP1 | MLP1S3 | High | Hill_E | 521 | 222703.69 | 3155160.60 | 2 | 60 |
| 2022-05-31 | LP | MLP2 | MLP2E1 | Low | Hill_E | 459 | 228518.05 | 3167096.86 | 4 | 55 |
| 2022-05-31 | LP | MLP2 | MLP2E2 | Low | Hill_E | 459 | 228516.24 | 3167096.17 | 4 | 56 |
| 2022-05-31 | LP | MLP2 | MLP2E3 | Low | Hill_E | 458 | 228513.46 | 3167086.89 | 4 | 57 |
| 2022-05-31 | LP | MLP2 | MLP2S1 | High | Hill_E | 456 | 228513.97 | 3167074.20 | 2 | 55 |
| 2022-05-31 | LP | MLP2 | MLP2S2 | High | Hill_E | 457 | 228513.49 | 3167077.08 | 2 | 56 |
| 2022-05-31 | LP | MLP2 | MLP2S3 | High | Hill_E | 460 | 228522.44 | 3167114.48 | 2 | 57 |
| 2022-05-31 | LP | MLP3 | MLP3E1 | Low | Coast_E | 38 | 230303.00 | 3171591.00 | 4 | 61 |
| 2022-05-31 | LP | MLP3 | MLP3E2 | Low | Coast_E | 38 | 230281.40 | 3171568.49 | 4 | 62 |
| 2022-05-31 | LP | MLP3 | MLP3E3 | Low | Coast_E | 33 | 230308.66 | 3171596.11 | 4 | 63 |
| 2022-05-31 | LP | MLP3 | MLP3S1 | High | Coast_E | 38 | 230277.92 | 3171592.15 | na | na |
| 2022-05-31 | LP | MLP3 | MLP3S2 | High | Coast_E | 33 | 230303.08 | 3171615.78 | na | na |
| 2022-05-31 | LP | MLP3 | MLP3S3 | High | Coast_E | 33 | 230305.10 | 3171619.43 | 2 | 63 |
| 2022-05-31 | LP | MLP4 | MLP4E1 | Low | Coast_E | 230 | 232397.20 | 3183404.53 | 4 | 64 |
| 2022-05-31 | LP | MLP4 | MLP4E2 | Low | Coast_E | 249 | 232300.28 | 3183440.07 | 4 | 65 |
| 2022-05-31 | LP | MLP4 | MLP4E3 | Low | Coast_E | 249 | 232297.97 | 3183438.89 | 4 | 66 |
| 2022-05-31 | LP | MLP4 | MLP4S1 | High | Coast_E | 227 | 232407.91 | 3183390.42 | 2 | 64 |
| 2022-05-31 | LP | MLP4 | MLP4S2 | High | Coast_E | 227 | 232407.91 | 3183390.42 | 2 | 65 |
| 2022-05-31 | LP | MLP4 | MLP4S3 | High | Coast_E | 227 | 232407.56 | 3183390.13 | 2 | 66 |
| 2022-06-01 | LP | MLP5 | MLP5E1 | Low | Hill_W | 342 | 211588.04 | 3191272.70 | 4 | 68 |
| 2022-06-01 | LP | MLP5 | MLP5E2 | Low | Hill_W | 342 | 211589.79 | 3191269.97 | 4 | 69 |
| 2022-06-01 | LP | MLP5 | MLP5E3 | Low | Hill_W | 353 | 211649.19 | 3191247.56 | 4 | 70 |
| 2022-06-01 | LP | MLP5 | MLP5S1 | High | Hill_W | 343 | 211582.68 | 3191283.14 | 2 | 68 |
| 2022-06-01 | LP | MLP5 | MLP5S2 | High | Hill_W | 343 | 211578.56 | 3191287.05 | 2 | 69 |
| 2022-06-01 | LP | MLP5 | MLP5S3 | High | Hill_W | 338 | 211611.08 | 3191258.09 | 2 | 70 |
| 2022-06-01 | LP | MLP6 | MLP6E1 | Low | Hill_W | 357 | 207412.00 | 3184592.00 | 4 | 71 |
| 2022-06-01 | LP | MLP6 | MLP6E2 | Low | Hill_W | 348 | 207436.74 | 3184544.99 | 4 | 72 |
| 2022-06-01 | LP | MLP6 | MLP6E3 | Low | Hill_W | 357 | 207407.00 | 3184592.00 | 4 | 73 |
| 2022-06-01 | LP | MLP6 | MLP6S1 | High | Hill_W | 340 | 207443.70 | 3184507.25 | 2 | 71 |
| 2022-06-01 | LP | MLP6 | MLP6S2 | High | Hill_W | 343 | 207444.91 | 3184519.91 | na | na |
| 2022-06-01 | LP | MLP6 | MLP6S3 | High | Hill_W | 352 | 207389.20 | 3184587.51 | 2 | 73 |
| 2022-06-01 | LP | MLP7 | MLP7E1 | Low | Hill_W | 679 | 211140.39 | 3180026.27 | 4 | 74 |
| 2022-06-01 | LP | MLP7 | MLP7E2 | Low | Hill_W | 683 | 211161.72 | 3179993.41 | 4 | 75 |
| 2022-06-01 | LP | MLP7 | MLP7E3 | Low | Hill_W | 683 | 211173.92 | 3180000.57 | 4 | 76 |
| 2022-06-01 | LP | MLP7 | MLP7S1 | High | Hill_W | 679 | 211176.96 | 3179958.80 | 2 | 74 |
| 2022-06-01 | LP | MLP7 | MLP7S2 | High | Hill_W | 675 | 211137.42 | 3179995.42 | 2 | 75 |
| 2022-06-01 | LP | MLP7 | MLP7S3 | High | Hill_W | 679 | 211141.47 | 3180028.06 | 2 | 76 |
| 2022-06-06 | LP | MLP8 | MLP8E1 | Low | Coast_W | 163 | 219006.02 | 3156839.9 | 4 | 77 |
| 2022-06-06 | LP | MLP8 | MLP8E2 | Low | Coast_W | 163 | 219005.76 | 3156828.81 | 3 | 78 |
| 2022-06-06 | LP | MLP8 | MLP8E3 | Low | Coast_W | 163 | 218995.17 | 3156795.79 | 4 | 79 |
| 2022-06-06 | LP | MLP8 | MLP8S1 | High | Coast_W | 163 | 218997.95 | 3156866.28 | 2 | 78 |
| 2022-06-06 | LP | MLP8 | MLP8S2 | High | Coast_W | 163 | 219006.02 | 3156839.9 | 2 | 79 |
| 2022-06-06 | LP | MLP8 | MLP8S3 | High | Coast_W | 163 | 219006.29 | 3156850.98 | na | na |
| 2022-06-06 | LP | MLP9 | MLP9E1 | Low | Hill_E | 508 | 227875 | 3164539 | 4 | 80 |
| 2022-06-06 | LP | MLP9 | MLP9E2 | Low | Hill_E | 508 | 227864 | 3164541 | 4 | 81 |
| 2022-06-06 | LP | MLP9 | MLP9E3 | Low | Hill_E | 508 | 227863 | 3164542 | 4 | 82 |
| 2022-06-06 | LP | MLP9 | MLP9S1 | High | Hill_E | 508 | 227898 | 3164527 | 2 | 81 |
| 2022-06-06 | LP | MLP9 | MLP9S2 | High | Hill_E | 508 | 227898 | 3164527 | 2 | 82 |
| 2022-06-06 | LP | MLP9 | MLP9S3 | High | Hill_E | 508 | 227876 | 3164540 | 2 | 83 |
| 2022-05-02 | TF | MTF12 | MTF12E1 | Low | Coast_S | 126 | 373670.00 | 3145703.00 | 3 | 39 |
| 2022-05-02 | TF | MTF12 | MTF12E2 | Low | Coast_S | 126 | 373635.00 | 3145706.00 | 3 | 40 |
| 2022-05-02 | TF | MTF12 | MTF12E3 | Low | Coast_S | 126 | 373639.00 | 3145703.00 | 3 | 41 |
| 2022-05-02 | TF | MTF12 | MTF12S1 | High | Coast_S | 126 | 373642.00 | 3145710.00 | 1 | 39 |
| 2022-05-02 | TF | MTF12 | MTF12S2 | High | Coast_S | 126 | 373656.53 | 3145715.18 | 1 | 40 |
| 2022-05-02 | TF | MTF12 | MTF12S3 | High | Coast_S | 126 | 373649.00 | 3145709.00 | 1 | 41 |
| 2022-05-05 | TF | MTF13 | MTF13E1 | Low | Hill_S | 586 | 368151.70 | 3145464.41 | 3 | 65 |
| 2022-05-05 | TF | MTF13 | MTF13E2 | Low | Hill_S | 586 | 368134.00 | 3145463.00 | 3 | 66 |
| 2022-05-05 | TF | MTF13 | MTF13E3 | Low | Hill_S | 586 | 368110.00 | 3145471.00 | 3 | 67 |
| 2022-05-05 | TF | MTF13 | MTF13S1 | High | Hill_S | 586 | 368121.00 | 3145471.00 | 1 | 65 |
| 2022-05-05 | TF | MTF13 | MTF13S2 | High | Hill_S | 586 | 368121.00 | 3145462.00 | 1 | 66 |
| 2022-05-05 | TF | MTF13 | MTF13S3 | High | Hill_S | 586 | 368101.00 | 3145471.00 | 1 | 67 |
| 2022-05-05 | TF | MTF15 | MTF15E1 | Low | Coast_S | 28 | 371015.00 | 3142835.73 | 3 | 68 |
| 2022-05-05 | TF | MTF15 | MTF15E2 | Low | Coast_S | 28 | 370996.00 | 3142844.00 | 3 | 69 |
| 2022-05-05 | TF | MTF15 | MTF15E3 | Low | Coast_S | 28 | 370996.00 | 3142852.00 | 3 | 70 |
| 2022-05-05 | TF | MTF15 | MTF15S1 | High | Coast_S | 28 | 370999.00 | 3142857.00 | na | na |
| 2022-05-05 | TF | MTF15 | MTF15S2 | High | Coast_S | 28 | 371004.00 | 3142861.00 | na | na |
| 2022-05-05 | TF | MTF15 | MTF15S3 | High | Coast_S | 28 | 371007.00 | 3142866.00 | na | na |
| 2022-05-02 | TF | MTF16 | MTF16E1 | Low | Hill_S | 377 | 365010.01 | 3140842.10 | 3 | 45 |
| 2022-05-02 | TF | MTF16 | MTF16E2 | Low | Hill_S | 377 | 365002.00 | 3140837.00 | 3 | 46 |
| 2022-05-02 | TF | MTF16 | MTF16E3 | Low | Hill_S | 377 | 365004.00 | 3140837.00 | 3 | 47 |
| 2022-05-02 | TF | MTF16 | MTF16S1 | High | Hill_S | 377 | 365005.00 | 3140835.00 | 1 | 45 |
| 2022-05-02 | TF | MTF16 | MTF16S2 | High | Hill_S | 377 | 365007.00 | 3140838.00 | 1 | 46 |
| 2022-05-02 | TF | MTF16 | MTF16S3 | High | Hill_S | 377 | 365008.00 | 3140836.00 | 1 | 47 |
| 2022-05-02 | TF | MTF18 | MTF18E1 | Low | Coast_S | 26 | 365873.00 | 3138184.00 | 3 | 42 |
| 2022-05-02 | TF | MTF18 | MTF18E2 | Low | Coast_S | 26 | 365865.00 | 3138188.00 | 3 | 43 |
| 2022-05-02 | TF | MTF18 | MTF18E3 | Low | Coast_S | 26 | 365857.00 | 3138189.00 | 3 | 44 |
| 2022-05-02 | TF | MTF18 | MTF18S1 | High | Coast_S | 26 | 365894.88 | 3138182.41 | na | na |
| 2022-05-02 | TF | MTF18 | MTF18S2 | High | Coast_S | 26 | 365883.00 | 3138185.00 | na | na |
| 2022-05-02 | TF | MTF18 | MTF18S3 | High | Coast_S | 26 | 365888.00 | 3138195.00 | 1 | 44 |
| 2022-05-03 | TF | MTF19 | MTF19E1 | Low | Hill_S | 581 | 361540.36 | 3137589.18 | 3 | 49 |
| 2022-05-03 | TF | MTF19 | MTF19E2 | Low | Hill_S | 581 | 361543.00 | 3137596.00 | 3 | 50 |
| 2022-05-03 | TF | MTF19 | MTF19E3 | Low | Hill_S | 581 | 361541.00 | 3137608.00 | 3 | 51 |
| 2022-05-03 | TF | MTF19 | MTF19S1 | High | Hill_S | 581 | 361547.00 | 3137595.00 | 1 | 49 |
| 2022-05-03 | TF | MTF19 | MTF19S2 | High | Hill_S | 581 | 361545.00 | 3137586.00 | 1 | 50 |
| 2022-05-03 | TF | MTF19 | MTF19S3 | High | Hill_S | 581 | 361547.00 | 3137583.00 | 1 | 51 |
| 2022-05-03 | TF | MTF20 | MTF20E1 | Low | Coast_S | 6 | 364470.00 | 3129839.00 | 3 | 61 |
| 2022-05-03 | TF | MTF20 | MTF20E2 | Low | Coast_S | 6 | 364447.00 | 3129876.00 | 3 | 62 |
| 2022-05-03 | TF | MTF20 | MTF20E3 | Low | Coast_S | 6 | 364446.00 | 3129855.00 | 3 | 63 |
| 2022-05-03 | TF | MTF20 | MTF20S1 | High | Coast_S | 6 | 364460.06 | 3129860.65 | 1 | 61 |
| 2022-05-03 | TF | MTF20 | MTF20S2 | High | Coast_S | 6 | 364473.00 | 3129856.00 | 1 | 62 |
| 2022-05-03 | TF | MTF20 | MTF20S3 | High | Coast_S | 6 | 364478.00 | 3129850.00 | 1 | 63 |
| 2022-05-03 | TF | MTF21 | MTF21S3 | High | Coast_S | 291 | 361210.00 | 3131847.00 | 1 | 54 |
| 2022-05-03 | TF | MTF21 | MTF21E1 | Low | Coast_S | 291 | 361186.00 | 3131841.00 | 3 | 52 |
| 2022-05-03 | TF | MTF21 | MTF21E2 | Low | Coast_S | 291 | 361194.00 | 3131828.00 | 3 | 53 |
| 2022-05-03 | TF | MTF21 | MTF21E3 | Low | Coast_S | 291 | 361201.00 | 3131829.00 | 3 | 54 |
| 2022-05-03 | TF | MTF21 | MTF21S1 | High | Coast_S | 291 | 361196.00 | 3131847.00 | 1 | 52 |
| 2022-05-03 | TF | MTF21 | MTF21S2 | High | Coast_S | 291 | 361203.00 | 3131846.00 | 1 | 53 |
| 2022-05-03 | TF | MTF23 | MTF23E1 | Low | Hill_S | 348 | 359324.00 | 3123633.00 | 3 | 55 |
| 2022-05-03 | TF | MTF23 | MTF23E2 | Low | Hill_S | 348 | 359321.00 | 3123645.00 | 3 | 56 |
| 2022-05-03 | TF | MTF23 | MTF23E3 | Low | Hill_S | 348 | 359324.00 | 3123643.00 | 3 | 57 |
| 2022-05-03 | TF | MTF23 | MTF23S1 | High | Hill_S | 348 | 359318.11 | 3123643.32 | 1 | 55 |
| 2022-05-03 | TF | MTF23 | MTF23S2 | High | Hill_S | 348 | 359318.00 | 3123634.00 | 1 | 56 |
| 2022-05-03 | TF | MTF23 | MTF23S3 | High | Hill_S | 348 | 359313.00 | 3123635.00 | 1 | 57 |
| 2022-05-03 | TF | MTF24 | MTF24E1 | Low | Coast_S | 47 | 361136.00 | 3122622.00 | 3 | 58 |
| 2022-05-03 | TF | MTF24 | MTF24E2 | Low | Coast_S | 47 | 361130.00 | 3122615.00 | 3 | 59 |
| 2022-05-03 | TF | MTF24 | MTF24E3 | Low | Coast_S | 47 | 361126.00 | 3122611.00 | 3 | 60 |
| 2022-05-03 | TF | MTF24 | MTF24S1 | High | Coast_S | 47 | 361119.00 | 3122600.00 | 1 | 58 |
| 2022-05-03 | TF | MTF24 | MTF24S2 | High | Coast_S | 47 | 361111.00 | 3122585.00 | na | na |
| 2022-05-03 | TF | MTF24 | MTF24S3 | High | Coast_S | 47 | 361105.00 | 3122579.00 | na | na |
| 2022-05-09 | TF | MTF28 | MTF28E1 | Low | Hill_S | 525 | 351762.72 | 3115601.76 | 3 | 72 |
| 2022-05-09 | TF | MTF28 | MTF28E2 | Low | Hill_S | 525 | 351763.47 | 3115601.76 | 3 | 73 |
| 2022-05-09 | TF | MTF28 | MTF28E3 | Low | Hill_S | 525 | 351764.49 | 3115599.08 | 3 | 74 |
| 2022-05-09 | TF | MTF28 | MTF28S1 | High | Hill_S | 525 | 351756.88 | 3115604.51 | 1 | 72 |
| 2022-05-09 | TF | MTF28 | MTF28S2 | High | Hill_S | 525 | 351756.80 | 3115608.71 | 1 | 73 |
| 2022-05-10 | TF | MTF28 | MTF28S3 | High | Hill_S | 525 | 351755.94 | 3115630.00 | 1 | 74 |
| 2022-05-09 | TF | MTF29 | MTF29E1 | Low | Coast_S | 20 | 356221.00 | 3111866.56 | 3 | 75 |
| 2022-05-09 | TF | MTF29 | MTF29E2 | Low | Coast_S | 20 | 356222.54 | 3111871.86 | 3 | 76 |
| 2022-05-09 | TF | MTF29 | MTF29E3 | Low | Coast_S | 20 | 356221.57 | 3111863.42 | 3 | 77 |
| 2022-05-09 | TF | MTF29 | MTF29S1 | High | Coast_S | 20 | 356255.63 | 3111853.06 | 1 | 75 |
| 2022-05-09 | TF | MTF29 | MTF29S2 | High | Coast_S | 20 | 356281.59 | 3111862.30 | 1 | 76 |
| 2022-05-09 | TF | MTF29 | MTF29S3 | High | Coast_S | 20 | 356260.08 | 3111856.94 | 1 | 77 |
| 2022-05-09 | TF | MTF32 | MTF32E1 | Low | Coast_S | 75 | 350988.37 | 3106950.22 | 3 | 81 |
| 2022-05-09 | TF | MTF32 | MTF32E2 | Low | Coast_S | 75 | 350952.32 | 3107025.20 | 3 | 82 |
| 2022-05-09 | TF | MTF32 | MTF32E3 | Low | Coast_S | 75 | 350976.71 | 3106962.90 | 3 | 83 |
| 2022-05-09 | TF | MTF32 | MTF32S1 | High | Coast_S | 75 | 350890.20 | 3107031.46 | na | na |
| 2022-05-09 | TF | MTF32 | MTF32S2 | High | Coast_S | 75 | 350901.59 | 3107026.81 | na | na |
| 2022-05-09 | TF | MTF32 | MTF32S3 | High | Coast_S | 75 | 350903.75 | 3107024.32 | 1 | 83 |
| 2022-05-09 | TF | MTF34 | MTF34E1 | Low | Coast_S | 5 | 344979.47 | 3101748.77 | 3 | 78 |
| 2022-05-09 | TF | MTF34 | MTF34E2 | Low | Coast_S | 5 | 344970.47 | 3101753.02 | 3 | 79 |
| 2022-05-09 | TF | MTF34 | MTF34E3 | Low | Coast_S | 5 | 344970.58 | 3101750.77 | 3 | 80 |
| 2022-05-09 | TF | MTF34 | MTF34S1 | High | Coast_S | 5 | 344984.54 | 3101747.26 | 1 | 78 |
| 2022-05-09 | TF | MTF34 | MTF34S2 | High | Coast_S | 5 | 344975.16 | 3101752.08 | 1 | 79 |
| 2022-05-09 | TF | MTF34 | MTF34S3 | High | Coast_S | 5 | 344965.19 | 3101757.37 | 1 | 80 |
| 2022-05-10 | TF | MTF37 | MTF37E1 | Low | Coast_S | 5 | 336719.43 | 3099420.04 | 3 | 88 |
| 2022-05-10 | TF | MTF37 | MTF37E2 | Low | Coast_S | 5 | 336732.07 | 3099411.32 | 3 | 89 |
| 2022-05-10 | TF | MTF37 | MTF37E3 | Low | Coast_S | 5 | 336733.07 | 3099428.12 | 3 | 90 |
| 2022-05-10 | TF | MTF37 | MTF37S1 | High | Coast_S | 5 | 336719.43 | 3099420.04 | na | na |
| 2022-05-10 | TF | MTF37 | MTF37S2 | High | Coast_S | 5 | 336720.75 | 3099419.09 | 1 | 89 |
| 2022-05-10 | TF | MTF37 | MTF37S3 | High | Coast_S | 5 | 336601.60 | 3099343.18 | na | na |
| 2022-05-10 | TF | MTF39 | MTF39E1 | Low | Coast_S | 300 | 334188.00 | 3106786.00 | 3 | 85 |
| 2022-05-10 | TF | MTF39 | MTF39E2 | Low | Coast_S | 300 | 334189.00 | 3106786.00 | 3 | 86 |
| 2022-05-10 | TF | MTF39 | MTF39E3 | Low | Coast_S | 300 | 334190.68 | 3106785.33 | 3 | 87 |
| 2022-05-10 | TF | MTF39 | MTF39S1 | High | Coast_S | 300 | 334185.00 | 3106771.02 | 1 | 85 |
| 2022-05-10 | TF | MTF39 | MTF39S2 | High | Coast_S | 300 | 334186.05 | 3106779.41 | 1 | 86 |
| 2022-05-10 | TF | MTF39 | MTF39S3 | High | Coast_S | 300 | 334189.25 | 3106777.86 | 1 | 87 |
| 2022-05-10 | TF | MTF40 | MTF40E1 | Low | Coast_S | 15 | 328065.43 | 3109451.31 | 3 | 94 |
| 2022-05-10 | TF | MTF40 | MTF40E2 | Low | Coast_S | 15 | 328067.33 | 3109453.91 | 3 | 95 |
| 2022-05-10 | TF | MTF40 | MTF40E3 | Low | Coast_S | 15 | 328062.00 | 3109450.00 | 3 | 96 |
| 2022-05-10 | TF | MTF40 | MTF40S1 | High | Coast_S | 15 | 328070.42 | 3109455.46 | 1 | 94 |
| 2022-05-10 | TF | MTF40 | MTF40S2 | High | Coast_S | 15 | 328064.79 | 3109456.81 | na | na |
| 2022-05-10 | TF | MTF40 | MTF40S3 | High | Coast_S | 15 | 328065.40 | 3109457.44 | na | na |
| 2022-05-10 | TF | MTF44 | MTF44E2 | Low | Hill_S | 660 | 325976.22 | 3122157.17 | 3 | 92 |
| 2022-05-10 | TF | MTF44 | MTF44E1 | Low | Hill_S | 660 | 325974.37 | 3122157.37 | 3 | 91 |
| 2022-05-10 | TF | MTF44 | MTF44E3 | Low | Hill_S | 660 | 325976.78 | 3122157.75 | 3 | 93 |
| 2022-05-10 | TF | MTF44 | MTF44S1 | High | Hill_S | 660 | 325975.67 | 3122156.09 | 1 | 91 |
| 2022-05-10 | TF | MTF44 | MTF44S2 | High | Hill_S | 660 | 325974.53 | 3122152.91 | 1 | 92 |
| 2022-05-10 | TF | MTF44 | MTF44S3 | High | Hill_S | 660 | 325973.40 | 3122149.55 | na | na |
| 2022-05-24 | TF | MTF46 | MTF46E1 | Low | Coast_S | 14 | 319895.56 | 3122570.16 | 4 | 51 |
| 2022-05-24 | TF | MTF46 | MTF46E2 | Low | Coast_S | 12 | 319887.00 | 3122561.90 | 4 | 52 |
| 2022-05-24 | TF | MTF46 | MTF46E3 | Low | Coast_S | 16 | 319907.01 | 3122561.60 | 4 | 53 |
| 2022-05-24 | TF | MTF46 | MTF46S1 | High | Coast_S | 15 | 319906.22 | 3122579.42 | na | na |
| 2022-05-24 | TF | MTF46 | MTF46S2 | High | Coast_S | 14 | 319898.58 | 3122561.73 | 2 | 52 |
| 2022-05-24 | TF | MTF46 | MTF46S3 | High | Coast_S | 13 | 319891.46 | 3122578.65 | 2 | 53 |
| 2022-05-24 | TF | MTF48 | MTF48E1 | Low | Hill_S | 815 | 322401.07 | 3129569.26 | 4 | 48 |
| 2022-05-24 | TF | MTF48 | MTF48E2 | Low | Hill_S | 811 | 322286.83 | 3129640.98 | 4 | 49 |
| 2022-05-24 | TF | MTF48 | MTF48E3 | Low | Hill_S | 812 | 322279.91 | 3129653.11 | 4 | 50 |
| 2022-05-24 | TF | MTF48 | MTF48S1 | High | Hill_S | 808 | 322285.69 | 3129617.41 | 2 | 48 |
| 2022-05-24 | TF | MTF48 | MTF48S2 | High | Hill_S | 811 | 322301.49 | 3129632.41 | na | na |
| 2022-05-24 | TF | MTF48 | MTF48S3 | High | Hill_S | 810 | 322265.66 | 3129652.39 | 2 | 50 |
| 2022-04-25 | TF | MTF4 | MTF4E1 | Low | Coast_S | 33 | 383354.00 | 3154177.00 | 3 | 35 |
| 2022-04-25 | TF | MTF4 | MTF4E2 | Low | Coast_S | 33 | 383352.00 | 3154175.00 | 3 | 36 |
| 2022-04-25 | TF | MTF4 | MTF4E3 | Low | Coast_S | 33 | 383358.00 | 3154179.00 | 3 | 37 |
| 2022-04-25 | TF | MTF4 | MTF4S1 | High | Coast_S | 33 | 383376.13 | 3154171.03 | na | na |
| 2022-04-25 | TF | MTF4 | MTF4S2 | High | Coast_S | 33 | 383359.00 | 3154175.00 | 1 | 36 |
| 2022-04-25 | TF | MTF4 | MTF4S3 | High | Coast_S | 33 | 383354.00 | 3154173.00 | na | na |
| 2022-05-23 | TF | MTF51 | MTF51E1 | Low | Coast_N | 157 | 320396.28 | 3139563.71 | 4 | 44 |
| 2022-05-23 | TF | MTF51 | MTF51E2 | Low | Coast_N | 157 | 320396.00 | 3139563.00 | 4 | 45 |
| 2022-05-23 | TF | MTF51 | MTF51E3 | Low | Coast_N | 147 | 320416.38 | 3139549.12 | 4 | 46 |
| 2022-05-23 | TF | MTF51 | MTF51S1 | High | Coast_N | 164 | 320409.10 | 3139587.44 | 2 | 44 |
| 2022-05-23 | TF | MTF51 | MTF51S2 | High | Coast_N | 164 | 320409.98 | 3139586.84 | na | na |
| 2022-05-23 | TF | MTF51 | MTF51S3 | High | Coast_N | 165 | 320411.72 | 3139589.40 | na | na |
| 2022-05-23 | TF | MTF52 | MTF52E1 | Low | Coast_N | 56 | 318127.03 | 3140563.68 | 4 | 41 |
| 2022-05-23 | TF | MTF52 | MTF52E2 | Low | Coast_N | 58 | 318133.37 | 3140555.17 | 4 | 42 |
| 2022-05-23 | TF | MTF52 | MTF52E3 | Low | Coast_N | 60 | 318155.81 | 3140558.87 | 4 | 43 |
| 2022-05-23 | TF | MTF52 | MTF52S1 | High | Coast_N | 52 | 318152.34 | 3140607.05 |  |  |
| 2022-05-23 | TF | MTF52 | MTF52S2 | High | Coast_N | 54 | 318146.85 | 3140591.44 |  |  |
| 2022-05-23 | TF | MTF52 | MTF52S3 | High | Coast_N | 51 | 318168.22 | 3140613.08 |  |  |
| 2022-05-17 | TF | MTF56 | MTF56E1 | Low | Coast_N | 10 | 330953.68 | 3140458.27 | 4 | 37 |
| 2022-05-17 | TF | MTF56 | MTF56E2 | Low | Coast_N | 10 | 330952.84 | 3140457.39 | 4 | 38 |
| 2022-05-17 | TF | MTF56 | MTF56E3 | Low | Coast_N | 10 | 330954.28 | 3140458.86 | 4 | 39 |
| 2022-05-17 | TF | MTF56 | MTF56S1 | High | Coast_N | 18 | 330980.14 | 3140472.06 | 2 | 37 |
| 2022-05-17 | TF | MTF56 | MTF56S2 | High | Coast_N | 13 | 330973.58 | 3140456.16 | 2 | 38 |
| 2022-05-17 | TF | MTF56 | MTF56S3 | High | Coast_N | 11 | 330947.85 | 3140449.19 | na | na |
| 2022-05-17 | TF | MTF57 | MTF57E1 | Low | Coast_N | 183 | 331100.02 | 3139314.63 | 4 | 34 |
| 2022-05-17 | TF | MTF57 | MTF57E2 | Low | Coast_N | 184 | 331102.29 | 3139305.20 | 4 | 35 |
| 2022-05-17 | TF | MTF57 | MTF57E3 | Low | Coast_N | 188 | 331107.65 | 3139275.67 | 4 | 36 |
| 2022-05-17 | TF | MTF57 | MTF57S1 | High | Coast_N | 183 | 331101.85 | 3139321.17 | 2 | 34 |
| 2022-05-17 | TF | MTF57 | MTF57S2 | High | Coast_N | 180 | 331091.82 | 3139339.14 | na | na |
| 2022-05-17 | TF | MTF57 | MTF57S3 | High | Coast_N | 180 | 331096.00 | 3139349.00 | 2 | 36 |
| 2022-05-17 | TF | MTF59 | MTF59E1 | Low | Coast_N | 181 | 334870.67 | 3140909.58 | 4 | 31 |
| 2022-05-17 | TF | MTF59 | MTF59E2 | Low | Coast_N | 181 | 334863.28 | 3140907.60 | 4 | 32 |
| 2022-05-17 | TF | MTF59 | MTF59E3 | Low | Coast_N | 181 | 334866.48 | 3140910.69 | 4 | 33 |
| 2022-05-17 | TF | MTF59 | MTF59S1 | High | Coast_N | 188 | 335009.05 | 3140953.69 | na | na |
| 2022-05-17 | TF | MTF59 | MTF59S2 | High | Coast_N | 186 | 334989.35 | 3140975.56 | 2 | 32 |
| 2022-05-17 | TF | MTF59 | MTF59S3 | High | Coast_N | 185 | 334990.83 | 3140987.45 | 2 | 33 |
| 2022-04-25 | TF | MTF5 | MTF5E1 | Low | Coast_S | 219 | 378912.00 | 3155268.00 | 3 | 32 |
| 2022-04-25 | TF | MTF5 | MTF5E2 | Low | Coast_S | 219 | 378929.00 | 3155270.00 | 3 | 33 |
| 2022-04-25 | TF | MTF5 | MTF5E3 | Low | Coast_S | 219 | 378921.20 | 3155271.70 | 3 | 34 |
| 2022-04-25 | TF | MTF5 | MTF5S1 | High | Coast_S | 219 | 378918.00 | 3155269.00 | 1 | 32 |
| 2022-04-25 | TF | MTF5 | MTF5S2 | High | Coast_S | 219 | 378921.20 | 3155271.70 | 1 | 33 |
| 2022-04-25 | TF | MTF5 | MTF5S3 | High | Coast_S | 219 | 378924.00 | 3155270.00 | 1 | 34 |
| 2022-05-16 | TF | MTF64 | MTF64E1 | Low | Hill_N | 371 | 338399.00 | 3140435.02 | 4 | 15 |
| 2022-05-16 | TF | MTF64 | MTF64E2 | Low | Hill_N | 370 | 338390.87 | 3140433.96 | 4 | 16 |
| 2022-05-16 | TF | MTF64 | MTF64E3 | Low | Hill_N | 371 | 338391.43 | 3140433.25 | 4 | 17 |
| 2022-05-16 | TF | MTF64 | MTF64S1 | High | Hill_N | 369 | 338361.61 | 3140411.59 | 2 | 15 |
| 2022-05-16 | TF | MTF64 | MTF64S2 | High | Hill_N | 369 | 338354.87 | 3140410.06 | 2 | 16 |
| 2022-05-16 | TF | MTF64 | MTF64S3 | High | Hill_N | 369 | 338363.79 | 3140419.05 | 2 | 17 |
| 2022-05-16 | TF | MTF65 | MTF65E1 | Low | Coast_N | 22 | 347426.72 | 3143831.38 | 4 | 18 |
| 2022-05-16 | TF | MTF65 | MTF65E2 | Low | Coast_N | 21 | 347403.88 | 3143830.58 | 4 | 19 |
| 2022-05-16 | TF | MTF65 | MTF65E3 | Low | Coast_N | 23 | 347421.31 | 3143823.72 | 4 | 20 |
| 2022-05-16 | TF | MTF65 | MTF65S1 | High | Coast_N | 22 | 347413.45 | 3143829.97 | 2 | 18 |
| 2022-05-16 | TF | MTF65 | MTF65S2 | High | Coast_N | 24 | 347438.21 | 3143823.94 | 2 | 19 |
| 2022-05-16 | TF | MTF65 | MTF65S3 | High | Coast_N | 19 | 347396.20 | 3143855.03 | 2 | 20 |
| 2022-05-17 | TF | MTF68 | MTF68E1 | Low | Coast_N | 177 | 353507.92 | 3146336.96 | 4 | 28 |
| 2022-05-17 | TF | MTF68 | MTF68E2 | Low | Coast_N | 177 | 353518.75 | 3146342.43 | 4 | 29 |
| 2022-05-17 | TF | MTF68 | MTF68E3 | Low | Coast_N | 175 | 353500.74 | 3146351.68 | 4 | 30 |
| 2022-05-17 | TF | MTF68 | MTF68S1 | High | Coast_N | 188 | 353581.85 | 3146299.08 | 2 | 28 |
| 2022-05-17 | TF | MTF68 | MTF68S2 | High | Coast_N | 187 | 353580.34 | 3146301.34 | 2 | 29 |
| 2022-05-17 | TF | MTF68 | MTF68S3 | High | Coast_N | 188 | 353583.07 | 3146296.92 | 2 | 30 |
| 2022-05-16 | TF | MTF69 | MTF69E1 | Low | Coast_N | 280 | 352192.23 | 3143668.47 | 4 | 24 |
| 2022-05-16 | TF | MTF69 | MTF69E2 | Low | Coast_N | 280 | 352192.81 | 3143669.27 | 4 | 25 |
| 2022-05-16 | TF | MTF69 | MTF69E3 | Low | Coast_N | 279 | 352190.64 | 3143670.67 | 4 | 26 |
| 2022-05-16 | TF | MTF69 | MTF69S1 | High | Coast_N | 277 | 352184.97 | 3143671.66 | 2 | 24 |
| 2022-05-16 | TF | MTF69 | MTF69S2 | High | Coast_N | 273 | 352183.21 | 3143648.02 | 2 | 25 |
| 2022-05-16 | TF | MTF69 | MTF69S3 | High | Coast_N | 273 | 352187.00 | 3143637.00 | 2 | 26 |
| 2022-04-25 | TF | MTF6 | MTF6E1 | Low | Coast_S | 37 | 379938.00 | 3153162.00 | 3 | 22 |
| 2022-04-25 | TF | MTF6 | MTF6E2 | Low | Coast_S | 37 | 379948.00 | 3153170.00 | 3 | 20 |
| 2022-04-25 | TF | MTF6 | MTF6E3 | Low | Coast_S | 37 | 379942.00 | 3153172.00 | 3 | 21 |
| 2022-04-25 | TF | MTF6 | MTF6S1 | High | Coast_S | 37 | 379943.00 | 3153168.00 | 1 | 20 |
| 2022-04-25 | TF | MTF6 | MTF6S2 | High | Coast_S | 37 | 379946.00 | 3153167.00 | 1 | 21 |
| 2022-04-25 | TF | MTF6 | MTF6S3 | High | Coast_S | 37 | 379938.00 | 3153170.00 | 1 | 22 |
| 2022-04-25 | TF | MTF7 | MTF7S1 | High | Coast_S | 163 | 376257.14 | 3153701.54 | 1 | 23 |
| 2022-04-25 | TF | MTF7 | MTF7S2 | High | Coast_S | 163 | 376255.00 | 3153701.00 | 1 | 24 |
| 2022-04-25 | TF | MTF7 | MTF7S3 | High | Coast_S | 163 | 376257.00 | 3153700.00 | 1 | 25 |
| 2022-04-25 | TF | MTF7 | MTF7E1 | Low | Coast_S | 163 | 376253.00 | 3153704.00 | 3 | 23 |
| 2022-04-25 | TF | MTF7 | MTF7E2 | Low | Coast_S | 163 | 376255.00 | 3153697.00 | 3 | 24 |
| 2022-04-25 | TF | MTF7 | MTF7E3 | Low | Coast_S | 163 | 376255.00 | 3153701.00 | 3 | 25 |
| 2022-05-16 | TF | MTF72 | MTF72E1 | Low | Hill_N | 300 | 358648.66 | 3149850.34 | 4 | 21 |
| 2022-05-16 | TF | MTF72 | MTF72E2 | Low | Hill_N | 300 | 358649.29 | 3149849.54 | 4 | 22 |
| 2022-05-16 | TF | MTF72 | MTF72E3 | Low | Hill_N | 301 | 358651.49 | 3149844.21 | 4 | 23 |
| 2022-05-16 | TF | MTF72 | MTF72S1 | High | Hill_N | 297 | 358679.16 | 3149882.55 | na | na |
| 2022-05-16 | TF | MTF72 | MTF72S2 | High | Hill_N | 296 | 358710.02 | 3149903.86 | 2 | 22 |
| 2022-05-16 | TF | MTF72 | MTF72S3 | High | Hill_N | 296 | 358708.52 | 3149900.64 | 2 | 23 |
| 2022-05-12 | TF | MTF74 | MTF74E1 | Low | Hill_N | 546 | 364975.81 | 3153826.93 | 4 | 11 |
| 2022-05-12 | TF | MTF74 | MTF74E2 | Low | Hill_N | 537 | 364981.15 | 3153877.51 | 4 | 12 |
| 2022-05-12 | TF | MTF74 | MTF74E3 | Low | Hill_N | 537 | 364978.59 | 3153887.79 | 4 | 13 |
| 2022-05-12 | TF | MTF74 | MTF74S1 | High | Hill_N | 546 | 364972.51 | 3153840.83 | 2 | 11 |
| 2022-05-12 | TF | MTF74 | MTF74S2 | High | Hill_N | 543 | 364975.34 | 3153850.79 | 2 | 12 |
| 2022-05-12 | TF | MTF74 | MTF74S3 | High | Hill_N | 540 | 364988.00 | 3153865.00 | 2 | 13 |
| 2022-05-12 | TF | MTF75 | MTF75E1 | Low | Coast_N | 36 | 363518.00 | 3157522.33 | 4 | 8 |
| 2022-05-12 | TF | MTF75 | MTF75E2 | Low | Coast_N | 36 | 363509.45 | 3157527.74 | 4 | 9 |
| 2022-05-12 | TF | MTF75 | MTF75E3 | Low | Coast_N | 35 | 363512.56 | 3157524.57 | 4 | 10 |
| 2022-05-12 | TF | MTF75 | MTF75S1 | High | Coast_N | 43 | 363545.08 | 3157496.56 | na | na |
| 2022-05-12 | TF | MTF75 | MTF75S2 | High | Coast_N | 39 | 363526.13 | 3157511.40 | na | na |
| 2022-05-12 | TF | MTF75 | MTF75S3 | High | Coast_N | 33 | 363472.68 | 3157487.80 | 2 | 10 |
| 2022-05-12 | TF | MTF77 | MTF77E1 | Low | Hill_N | 516 | 370245.61 | 3156325.99 | 4 | 5 |
| 2022-05-12 | TF | MTF77 | MTF77E2 | Low | Hill_N | 512 | 370212.61 | 3156323.01 | 4 | 6 |
| 2022-05-12 | TF | MTF77 | MTF77E3 | Low | Hill_N | 502 | 370170.77 | 3156341.23 | na | na |
| 2022-05-12 | TF | MTF77 | MTF77S1 | High | Hill_N | 518 | 370256.95 | 3156328.43 | 2 | 5 |
| 2022-05-12 | TF | MTF77 | MTF77S2 | High | Hill_N | 505 | 370178.85 | 3156330.82 | 2 | 6 |
| 2022-05-12 | TF | MTF77 | MTF77S3 | High | Hill_N | 500 | 370159.00 | 3156345.00 | 2 | 7 |
| 2022-04-25 | TF | MTF8 | MTF8S1 | High | Coast_S | 19 | 378217.65 | 3151189.61 | 1 | 26 |
| 2022-04-25 | TF | MTF8 | MTF8S2 | High | Coast_S | 19 | 378216.93 | 3151188.47 | 1 | 27 |
| 2022-04-25 | TF | MTF8 | MTF8S3 | High | Coast_S | 19 | 378208.55 | 3151197.21 | 1 | 28 |
| 2022-04-25 | TF | MTF8 | MTF8E1 | Low | Coast_S | 19 | 378208.40 | 3151191.35 | 3 | 26 |
| 2022-04-25 | TF | MTF8 | MTF8E2 | Low | Coast_S | 19 | 378200.41 | 3151196.08 | 3 | 27 |
| 2022-04-25 | TF | MTF8 | MTF8E3 | Low | Coast_S | 19 | 378202.72 | 3151190.49 | 3 | 28 |
| 2022-04-25 | TF | MTF9 | MTF9S1 | High | Hill_S | 534 | 371697.59 | 3151624.83 | 1 | 29 |
| 2022-04-25 | TF | MTF9 | MTF9S2 | High | Hill_S | 534 | 371714.00 | 3151612.00 | 1 | 30 |
| 2022-04-25 | TF | MTF9 | MTF9S3 | High | Hill_S | 534 | 371698.00 | 3151612.00 | 1 | 31 |
| 2022-04-25 | TF | MTF9 | MTF9E1 | Low | Hill_S | 534 | 371706.00 | 3151609.00 | 3 | 29 |
| 2022-04-25 | TF | MTF9 | MTF9E2 | Low | Hill_S | 534 | 371721.00 | 3151608.00 | 3 | 30 |
| 2022-04-25 | TF | MTF9 | MTF9E3 | Low | Hill_S | 534 | 371713.00 | 3151607.00 | 3 | 31 |

*Coordinates are WGS84/UTM zone 28N (zone letter R)

| Table S8 Site × host-condition pooled groups used for rarefaction (Tenerife and La Palma) | | | |  |
| --- | --- | --- | --- | --- |
| Island | Group ID | Total sequences | Observed genera | |
| TF | TF_MTF12_High | 21 | 7 | |
| TF | TF_MTF12_Low | 90 | 11 | |
| TF | TF_MTF13_High | 115 | 19 | |
| TF | TF_MTF13_Low | 170 | 22 | |
| TF | TF_MTF15_Low | 235 | 19 | |
| TF | TF_MTF16_High | 28 | 5 | |
| TF | TF_MTF16_Low | 147 | 18 | |
| TF | TF_MTF18_Low | 87 | 9 | |
| TF | TF_MTF19_High | 28 | 8 | |
| TF | TF_MTF19_Low | 183 | 18 | |
| TF | TF_MTF20_High | 14 | 5 | |
| TF | TF_MTF20_Low | 128 | 5 | |
| TF | TF_MTF21_High | 59 | 12 | |
| TF | TF_MTF21_Low | 160 | 22 | |
| TF | TF_MTF23_High | 56 | 22 | |
| TF | TF_MTF23_Low | 173 | 21 | |
| TF | TF_MTF24_Low | 107 | 2 | |
| TF | TF_MTF28_High | 71 | 21 | |
| TF | TF_MTF28_Low | 176 | 27 | |
| TF | TF_MTF29_Low | 105 | 20 | |
| TF | TF_MTF32_Low | 97 | 9 | |
| TF | TF_MTF34_High | 159 | 12 | |
| TF | TF_MTF34_Low | 179 | 16 | |
| TF | TF_MTF37_Low | 88 | 13 | |
| TF | TF_MTF39_High | 26 | 5 | |
| TF | TF_MTF39_Low | 146 | 21 | |
| TF | TF_MTF4_Low | 81 | 9 | |
| TF | TF_MTF40_Low | 142 | 7 | |
| TF | TF_MTF44_Low | 185 | 23 | |
| TF | TF_MTF46_Low | 108 | 9 | |
| TF | TF_MTF48_Low | 145 | 16 | |
| TF | TF_MTF5_High | 44 | 10 | |
| TF | TF_MTF5_Low | 241 | 23 | |
| TF | TF_MTF51_Low | 232 | 24 | |
| TF | TF_MTF52_Low | 138 | 12 | |
| TF | TF_MTF56_Low | 126 | 10 | |
| TF | TF_MTF57_High | 35 | 15 | |
| TF | TF_MTF57_Low | 191 | 22 | |
| TF | TF_MTF59_Low | 223 | 19 | |
| TF | TF_MTF6_High | 45 | 8 | |
| TF | TF_MTF6_Low | 116 | 21 | |
| TF | TF_MTF64_High | 63 | 13 | |
| TF | TF_MTF64_Low | 104 | 17 | |
| TF | TF_MTF65_High | 57 | 14 | |
| TF | TF_MTF65_Low | 186 | 21 | |
| TF | TF_MTF68_High | 118 | 27 | |
| TF | TF_MTF68_Low | 92 | 17 | |
| TF | TF_MTF69_High | 27 | 8 | |
| TF | TF_MTF69_Low | 226 | 28 | |
| TF | TF_MTF7_High | 42 | 10 | |
| TF | TF_MTF7_Low | 144 | 23 | |
| TF | TF_MTF72_High | 56 | 17 | |
| TF | TF_MTF72_Low | 222 | 27 | |
| TF | TF_MTF74_High | 121 | 29 | |
| TF | TF_MTF74_Low | 83 | 24 | |
| TF | TF_MTF75_Low | 108 | 7 | |
| TF | TF_MTF77_High | 68 | 16 | |
| TF | TF_MTF77_Low | 144 | 29 | |
| TF | TF_MTF8_High | 48 | 13 | |
| TF | TF_MTF8_Low | 294 | 36 | |
| TF | TF_MTF9_High | 128 | 29 | |
| TF | TF_MTF9_Low | 160 | 21 | |
| LP | LP_MLP1_High | 211 | 41 | |
| LP | LP_MLP1_Low | 297 | 29 | |
| LP | LP_MLP10_Low | 201 | 24 | |
| LP | LP_MLP11_High | 21 | 9 | |
| LP | LP_MLP11_Low | 171 | 18 | |
| LP | LP_MLP12_High | 18 | 5 | |
| LP | LP_MLP12_Low | 214 | 25 | |
| LP | LP_MLP13_Low | 110 | 6 | |
| LP | LP_MLP2_High | 193 | 24 | |
| LP | LP_MLP2_Low | 250 | 20 | |
| LP | LP_MLP3_High | 30 | 7 | |
| LP | LP_MLP3_Low | 89 | 12 | |
| LP | LP_MLP4_High | 25 | 10 | |
| LP | LP_MLP4_Low | 162 | 14 | |
| LP | LP_MLP5_High | 96 | 22 | |
| LP | LP_MLP5_Low | 232 | 21 | |
| LP | LP_MLP6_High | 11 | 6 | |
| LP | LP_MLP6_Low | 298 | 23 | |
| LP | LP_MLP7_High | 12 | 5 | |
| LP | LP_MLP7_Low | 136 | 9 | |
| LP | LP_MLP8_Low | 92 | 21 | |
| LP | LP_MLP9_High | 134 | 33 | |
| LP | LP_MLP9_Low | 205 | 32 | |

**Rarefaction curves were computed for pooled site × host-condition groups; groups were retained when they contained ≥10 genus-assigned OTU records and ≥2 genera (LP: 23/26 groups retained; TF: 62/76 retained).”

**Fig. S2** Rarefaction curves for pooled site × host condition groups in Tenerife (TF) and La Palma(LP).

Curves were computed from at genus level from SCATA-derived read outputs after clustering and taxonomic curation, retaining only OTUs with a confirmed genus assignment and pooling OTUs by genus. For each site, genus counts were summed across the three plants within each host-condition class to generate a pooled community table. The x-axis shows the total number of genus-assigned OTU records per pooled group (plotting inclusion threshold: ≥10), and the y-axis shows observed genus richness (≥2). Curves are presented as a diagnostic of sequencing-depth heterogeneity and residual genus accumulation, not as evidence of complete sampling

| Table S9 FUNGuild annotation coverage and final guild-class distribution for retained genera in Tenerife (TF) and La Palma (LP) | | | | | | |
| --- | --- | --- | --- | --- | --- | --- |
| Panel A. Annotation coverage | | | | | | |
| Island | Retained genera (n) | Genera in FUNGuild file | Missing from FUNGuild file | Unassigned genera | Conflicting assignments within island (n) | Assignment confidence retained |
| TF | 184 | 184 | 0 | 12 | 0 | 172 |
| LP | 126 | 126 | 0 | 12 | 0 | 114 |

| Panel B. Final guild-class distribution | | |
| --- | --- | --- |
| Island | Guild class | Retained genera (n) |
| LP | Ectomycorrhizal | 1 |
| LP | Endophyte | 15 |
| LP | Other_pathotrophs | 10 |
| LP | Plant_pathogen | 35 |
| LP | Saprotroph | 53 |
| LP | Unassigned | 12 |
| TF | Ectomycorrhizal | 1 |
| TF | Endophyte | 13 |
| TF | Other_pathotrophs | 14 |
| TF | Plant_pathogen | 66 |
| TF | Saprotroph | 78 |
| TF | Unassigned | 12 |

| Panel C. Retained taxa without guild assignment | |  |
| --- | --- | --- |
| Genus | Island | |
| *Cippumomyces* | TF, LP | |
| *Gobabebomyces* | TF, LP | |
| *Heterospora* | TF, LP | |
| *Lembosiniella* | TF, LP | |
| *Longiseptatispora* | TF | |
| *Neoacrodontiella* | TF, LP | |
| *Nothophaeotheca* | TF | |
| *Paradevriesia* | TF, LP | |
| *Populomyces* | TF | |
| *Pseudocyclothyriella* | TF | |
| *Salinomyces* | TF | |
| *Vanderaaea* | TF, LP | |
| *Basidiodesertica* | LP | |
| *Dimorphoma* | LP | |
| *Golubevia* | LP | |
| *Marantokordyana* | LP | |
| *Pseudocyclothyriella* | LP | |

| Table S10 Sample sizes and genus counts for each analysis of beta diversity | | | | | | |
| --- | --- | --- | --- | --- | --- | --- |
| Analysis block | Island | Representation | Unit of analysis | No. of samples | No of sites | No of genera |
| PERMANOVA | TF | genus-count | plant | 204 | 39 | 184 |
| Mantel | TF | Bray-Curtis on genus count matrix + binary model distances | plant | 204 | 39 | 184 |
| NMDS | TF | genus-count | plant | 203 | 39 | 184 |
| RC_bray | TF | presence/absence | plant | 204 | 39 | 184 |
| VPA | TF | genus-count | plant | 204 | 39 | 184 |
| PERMANOVA | LP | genus-count | plant | 69 | 13 | 126 |
| Mantel | LP | Bray-Curtis on genus count matrix + binary model distances | plant | 69 | 13 | 126 |
| NMDS | LP | genus-count | plant | 68 | 13 | 126 |
| RC_bray | LP | presence/absence | plant | 69 | 13 | 126 |
| VPA | LP | genus-count | plant | 69 | 13 | 126 |

| Table S11 Genera calculated with indicator species analysis grouped within zone x host condition, per island. Values represent: A – specificity (probability that a genus belongs to the group), B – fidelity (probability of finding the genera in sites belonging to the group), stat – InDVal.g (overall indicator, geometric mean of A and B) and assigned ecological guilds. Only genera with p < 0.05 based on permutations are shown as ISA. | | | | | | |
| --- | --- | --- | --- | --- | --- | --- |
| Genus | Zone x host condition | Island | A | B | Stat | Guild |
| *Trimmatostroma* | Coast_N x High | TF | 0.67 | 0.4 | 0.52 | Other Pathotroph |
| *Toxicocladosporium* | Coast_N x High | TF | 0.30 | 0.78 | 0.48 | Endophyte |
| *Verrucocladosporium* | Coast_N x High | TF | 0.39 | 0.48 | 0.43 | Other Pathotroph |
| *Hortaea* | Coast_N x High | TF | 0.41 | 0.37 | 0.39 | Plant Pathogen |
| *Didymocyrtis* | Coast_N x High | TF | 0.40 | 0.33 | 0.36 | Plant Pathogen |
| *Stenella* | Coast_S x Low | TF | 0.77 | 0.33 | 0.51 | Plant Pathogen |
| *Eupenidiella* | Coast_S x Low | TF | 0.48 | 0.46 | 0.48 | Other Pathotroph |
| *Ramimonilia* | Coast_S x Low | TF | 0.64 | 0.22 | 0.38 | Saprotroph |
| *Symmetrospora* | Hill_N x High | TF | 0.37 | 0.91 | 0.58 | Saprotroph |
| *Erythrobasidium* | Hill_N x High | TF | 0.86 | 0.36 | 0.56 | Saprotroph |
| *Filobasidium* | Hill_N x High | TF | 0.32 | 0.82 | 0.51 | Saprotroph |
| *Vishniacozyma* | Hill_N x High | TF | 0.34 | 0.73 | 0.5 | Saprotroph |
| *Dioszegia* | Hill_N x High | TF | 0.37 | 0.55 | 0.45 | Saprotroph |
| *Kondoa* | Hill_N x High | TF | 0.44 | 0.45 | 0.45 | Saprotroph |
| *Xenoramularia* | Hill_N x High | TF | 1 | 0.19 | 0.43 | Plant Pathogen |
| *Kalmanozyma* | Hill_N x High | TF | 0.8 | 0.18 | 0.38 | Saprotroph |
| *Allophoma* | Hill_N x Low | TF | 0.82 | 0.36 | 0.55 | Plant Pathogen |
| *Phaeosphaeria* | Hill_N x Low | TF | 0.23 | 0.82 | 0.49 | Plant Pathogen |
| *Didymella* | Hill_N x Low | TF | 0.27 | 0.73 | 0.45 | Plant Pathogen |
| *Nigrospora* | Hill_N x Low | TF | 0.40 | 0.45 | 0.43 | Saprotroph |
| *Neodevriesia* | Hill_N x Low | TF | 0.49 | 0.36 | 0.42 | Plant Pathogen |
| *Nothophoma* | Hill_N x Low | TF | 0.37 | 0.45 | 0.41 | Saprotroph |
| *Paraphaeosphaeria* | Hill_N x Low | TF | 0.56 | 0.27 | 0.39 | Saprotroph |
| *Neodidymelliopsis* | Hill_N x Low | TF | 0.8 | 0.18 | 0.38 | Saprotroph |
| *Parastagonospora* | Hill_N x Low | TF | 0.47 | 0.27 | 0.36 | Plant Pathogen |
| *Epicoccum* | Hill_N x Low | TF | 0.34 | 0.36 | 0.35 | Endophyte |
| *Calophoma* | Hill_N x Low | TF | 0.56 | 0.18 | 0.32 | Plant Pathogen |
| *Cryptococcus* | Hill_S x High | TF | 1 | 0.09 | 0.30 | Plant Pathogen |
| *Davidiellomyces* | Hill_S x Low | TF | 0.45 | 0.38 | 0.41 | Saprotroph |
| *Pseudoseptoria* | Hill_S x Low | TF | 0.37 | 0.46 | 0.41 | Plant Pathogen |
| *Hormonema* | Hill_S x Low | TF | 0.20 | 0.83 | 0.41 | Saprotroph |
| *Staurosphaeria* | Hill_S x Low | TF | 0.63 | 0.25 | 0.4 | Saprotroph |
| *Neostagonospora* | Hill_S x Low | TF | 0.53 | 0.29 | 0.39 | Saprotroph |
| *Gobabebomyces* | Hill_S x Low | TF | 0.9 | 0.17 | 0.39 | na |
| *Selenophoma* | Hill_S x Low | TF | 0.35 | 0.38 | 0.36 | Saprotroph |
| *Saxophila* | Hill_S x Low | TF | 1 | 0.13 | 0.35 | Saprotroph |
| *Buckleyzyma* | Coast_E x High | LP | 0.55 | 0.4 | 0.47 | Saprotroph |
| *Phaeococcomyces* | Coast_E x Low | LP | 0.58 | 0.89 | 0.72 | Saprotroph |
| *Vanderaaea* | Coast_E x Low | LP | 0.77 | 0.56 | 0.65 | na |
| *Ramimonilia* | Coast_E x Low | LP | 0.78 | 0.44 | 0.59 | Saprotroph |
| *Cippumomyces* | Coast_E x Low | LP | 1 | 0.33 | 0.58 | na |
| *Staurosphaeria* | Coast_E x Low | LP | 0.8 | 0.33 | 0.52 | Saprotroph |
| *Hormonema* | Coast_E x Low | LP | 0.22 | 1 | 0.47 | Saprotroph |
| *Didymocyrtis* | Coast_W x Low | LP | 0.53 | 0.75 | 0.63 | Plant Pathogen |
| *Comoclathris* | Coast_W x Low | LP | 0.69 | 0.38 | 0.51 | Saprotroph |
| *Stagonosporopsis* | Coast_W x Low | LP | 1 | 0.25 | 0.5 | Plant Pathogen |
| *Neosetophoma* | Coast_W x Low | LP | 0.53 | 0.38 | 0.45 | Endophyte |
| *Hannaella* | Hill_E x High | LP | 1 | 0.33 | 0.58 | Other Pathotroph |
| *Vishniacozyma* | Hill_E x High | LP | 0.44 | 0.67 | 0.54 | Saprotroph |
| *Genolevuria* | Hill_E x High | LP | 1 | 0.25 | 0.5 | Saprotroph |
| *Symmetrospora* | Hill_E x High | LP | 0.32 | 0.67 | 0.47 | Saprotroph |
| *Selenophoma* | Hill_E x Low | LP | 0.59 | 0.83 | 0.7 | Saprotroph |
| *Neodevriesia* | Hill_E x Low | LP | 0.83 | 0.42 | 0.59 | Plant Pathogen |
| *Phaeosphaeria* | Hill_E x Low | LP | 0.4 | 0.67 | 0.51 | Endophyte |
| *Pseudoseptoria* | Hill_E x Low | LP | 0.35 | 0.75 | 0.51 | Plant Pathogen |
| *Cladosporium* | Hill_E x Low | LP | 0.26 | 1 | 0.51 | Plant Pathogen |
| *Toxicocladosporium* | Hill_E x Low | LP | 0.25 | 1 | 0.5 | Endophyte |
| *Neostagonospora* | Hill_E x Low | LP | 0.67 | 0.33 | 0.47 | Saprotroph |
| *Verrucocladosporium* | Hill_E x Low | LP | 0.38 | 0.58 | 0.47 | Other Pathotroph |
| *Alternaria* | Hill_W x Low | LP | 0.25 | 0.89 | 0.47 | Plant Pathogen |

| Table S12 Shared and host condition-specific genera (excluding the < 2% and < 3% for Tenerife and La Palma). For each island, genera are classified as shared between high and low-condition plants, or detected in one host condition category. | | |
| --- | --- | --- |
| Island | Host condition | Genera |
| TF | High & Low | *Alternaria* |
| TF | High & Low | *Aureobasidium* |
| TF | High & Low | *Cladosporium* |
| TF | High & Low | *Didymella* |
| TF | High & Low | *Epicoccum* |
| TF | High & Low | *Eupenidiella* |
| TF | High & Low | *Hormonema* |
| TF | High & Low | *Phaeosphaeria* |
| TF | High & Low | *Symmetrospora* |
| TF | High & Low | *Toxicocladosporium* |
| TF | High & Low | *Verrucocladosporium* |
| TF | High & Low | *Vishniacozyma* |
| TF | High | *Buckleyzyma* |
| TF | High | *Cutaneotrichosporon* |
| TF | High | *Dioszegia* |
| TF | High | *Erythrobasidium* |
| TF | High | *Filobasidium* |
| TF | High | *Hannaella* |
| TF | High | *Kondoa* |
| TF | High | *Papiliotrema* |
| TF | Low | *Allophoma* |
| TF | Low | *Didymocyrtis* |
| TF | Low | *Hortaea* |
| TF | Low | *Neodevriesia* |
| TF | Low | *Nigrospora* |
| TF | Low | *Nothophoma* |
| TF | Low | *Paraphaeosphaeria* |
| TF | Low | *Parastagonospora* |
| TF | Low | *Pseudoseptoria* |
| TF | Low | *Ramimonilia* |
| TF | Low | *Selenophoma* |
| TF | Low | *Stenella* |
| TF | Low | *Trimmatostroma* |
| LP | High & Low | *Alternaria* |
| LP | High & Low | *Aureobasidium* |
| LP | High & Low | *Cladosporium* |
| LP | High & Low | *Didymella* |
| LP | High & Low | *Epicoccum* |
| LP | High & Low | *Hormonema* |
| LP | High & Low | *Phaeosphaeria* |
| LP | High & Low | *Pseudoseptoria* |
| LP | High & Low | *Stemphylium* |
| LP | High & Low | *Toxicocladosporium* |
| LP | High & Low | *Vishniacozyma* |
| LP | High | *Apiospora* |
| LP | High | *Buckleyzyma* |
| LP | High | *Dioszegia* |
| LP | High | *Erythrobasidium* |
| LP | High | *Filobasidium* |
| LP | High | *Nigrospora* |
| LP | High | *Papiliotrema* |
| LP | High | *Paraloratospora* |
| LP | High | *Symmetrospora* |
| LP | Low | *Cippumomyces* |
| LP | Low | *Comoclathris* |
| LP | Low | *Didymocyrtis* |
| LP | Low | *Neosetophoma* |
| LP | Low | *Notophoma* |
| LP | Low | *Phaeococcomyces* |
| LP | Low | *Ramimonilia* |
| LP | Low | *Selenophoma* |
| LP | Low | *Staurosphaeria* |
| LP | Low | *Trimmatostroma* |
| LP | Low | *Vanderaaea* |
| LP | Low | *Verrucocladosporium* |

| Table S13 Numerical values underlying Figure 4: functional guild composition of retained fungal genera by island, environmental zone and host condition | | | | |
| --- | --- | --- | --- | --- |
| Island | Zone | Host Condition | Guild | Pie % |
| LP | Coast East | High | Endophyte | 16.67 |
| LP | Coast East | High | Other_pathotrophs | 4.17 |
| LP | Coast East | High | Plant_pathogen | 33.33 |
| LP | Coast East | High | Saprotroph | 45.83 |
| LP | Coast East | Low | Endophyte | 16.25 |
| LP | Coast East | Low | Other_pathotrophs | 1.25 |
| LP | Coast East | Low | Plant_pathogen | 25.00 |
| LP | Coast East | Low | Saprotroph | 42.50 |
| LP | Coast East | Low | Unassigned | 15.00 |
| LP | Coast West | High | Endophyte | 7.69 |
| LP | Coast West | High | Plant_pathogen | 61.54 |
| LP | Coast West | High | Saprotroph | 30.77 |
| LP | Coast West | Low | Ectomycorrhizal | 1.30 |
| LP | Coast West | Low | Endophyte | 23.38 |
| LP | Coast West | Low | Other_pathotrophs | 11.69 |
| LP | Coast West | Low | Plant_pathogen | 29.87 |
| LP | Coast West | Low | Saprotroph | 32.47 |
| LP | Coast West | Low | Unassigned | 1.30 |
| LP | Hill East | High | Endophyte | 14.20 |
| LP | Hill East | High | Other_pathotrophs | 8.64 |
| LP | Hill East | High | Plant_pathogen | 30.25 |
| LP | Hill East | High | Saprotroph | 44.44 |
| LP | Hill East | High | Unassigned | 2.47 |
| LP | Hill East | Low | Endophyte | 16.57 |
| LP | Hill East | Low | Other_pathotrophs | 5.71 |
| LP | Hill East | Low | Plant_pathogen | 38.29 |
| LP | Hill East | Low | Saprotroph | 36.57 |
| LP | Hill East | Low | Unassigned | 2.86 |
| LP | Hill West | High | Endophyte | 2.50 |
| LP | Hill West | High | Other_pathotrophs | 15.00 |
| LP | Hill West | High | Plant_pathogen | 50.00 |
| LP | Hill West | High | Saprotroph | 30.00 |
| LP | Hill West | High | Unassigned | 2.50 |
| LP | Hill West | Low | Endophyte | 8.99 |
| LP | Hill West | Low | Other_pathotrophs | 10.11 |
| LP | Hill West | Low | Plant_pathogen | 46.07 |
| LP | Hill West | Low | Saprotroph | 33.71 |
| LP | Hill West | Low | Unassigned | 1.12 |
| TF | Coast North | High | Endophyte | 8.41 |
| TF | Coast North | High | Other_pathotrophs | 6.54 |
| TF | Coast North | High | Plant_pathogen | 42.99 |
| TF | Coast North | High | Saprotroph | 42.06 |
| TF | Coast North | Low | Endophyte | 15.89 |
| TF | Coast North | Low | Other_pathotrophs | 12.02 |
| TF | Coast North | Low | Plant_pathogen | 39.15 |
| TF | Coast North | Low | Saprotroph | 31.78 |
| TF | Coast North | Low | Unassigned | 1.16 |
| TF | Coast South | High | Endophyte | 6.76 |
| TF | Coast South | High | Other_pathotrophs | 15.54 |
| TF | Coast South | High | Plant_pathogen | 43.92 |
| TF | Coast South | High | Saprotroph | 33.78 |
| TF | Coast South | Low | Endophyte | 11.68 |
| TF | Coast South | Low | Other_pathotrophs | 11.45 |
| TF | Coast South | Low | Plant_pathogen | 40.42 |
| TF | Coast South | Low | Saprotroph | 33.18 |
| TF | Coast South | Low | Unassigned | 3.27 |
| TF | Hill North | High | Endophyte | 8.11 |
| TF | Hill North | High | Other_pathotrophs | 2.70 |
| TF | Hill North | High | Plant_pathogen | 33.33 |
| TF | Hill North | High | Saprotroph | 55.86 |
| TF | Hill North | Low | Ectomycorrhizal | 0.74 |
| TF | Hill North | Low | Endophyte | 13.97 |
| TF | Hill North | Low | Other_pathotrophs | 0.74 |
| TF | Hill North | Low | Plant_pathogen | 38.97 |
| TF | Hill North | Low | Saprotroph | 43.38 |
| TF | Hill North | Low | Unassigned | 2.21 |
| TF | Hill South | High | Endophyte | 8.33 |
| TF | Hill South | High | Other_pathotrophs | 4.49 |
| TF | Hill South | High | Plant_pathogen | 42.95 |
| TF | Hill South | High | Saprotroph | 43.59 |
| TF | Hill South | High | Unassigned | 0.64 |
| TF | Hill South | Low | Endophyte | 12.79 |
| TF | Hill South | Low | Other_pathotrophs | 5.43 |
| TF | Hill South | Low | Plant_pathogen | 39.15 |
| TF | Hill South | Low | Saprotroph | 39.92 |
| TF | Hill South | Low | Unassigned | 2.71 |
| *** values correspond to the proportions used to generate the pie charts in Fig. 4. Percentages were calculated within each island × zone × host-condition group and sum to 100% per pie. Guilds were assigned using FUNGuild from the full retained genus matrix prior to any graphical aggregation. Unassigned includes retained genera lacking assignment in the database. | | | | |

| Table S14 Diversity indices Shannon and Hill0 values per group – island (TF – Tenerife, LP – La Palma), host condition level (high- or low-) and orientation (TF: N/S, LP: E/W). Lowercase letters indicate significant differences between groups within island (post hoc tests with *p < 0.05*). The post hoc test used in indicated in brackets: D = Dunn’s test with Bonferroni correction; T = Tukey’s HSD test. Groups are: i) coast versus hill of the same orientation, ii) same host condition level between hill vs. coast, iii) plant host low- versus high-condition. | | | |  |
| --- | --- | --- | --- | --- |
| Island | Group | Shannon | Hill0 | |
| LP | Coast_E | 2.4 a (T) | 40 a (D) | |
| LP | Coast_W | 2.8 ab (T) | 40 a (D) | |
| LP | Hill_E | 3.2 c (T) | 88 c (D) | |
| LP | Hill_W | 2.3 a (T) | 41 ab (D) | |
| LP | High | 3.3 a (T) | 76 a (D) | |
| LP | Low | 3 b (T) | 94 b (D) | |
| LP | High_Coast_E | 2.2 a (T) | 14 a (D) | |
| LP | High_Coast_W | 1.6 a (T) | 0.3 (D) | |
| LP | High_Hill_E | 3.3 d (T) | 64 d (D) | |
| LP | High_Hill_W | 2.4 ab (T) | 26 b (D) | |
| LP | Low_Coast_E | 2.3 ab (T) | 33 bc (D) | |
| LP | Low_Coast_W | 2.8 bc (T) | 38 c (D) | |
| LP | Low_Hill_E | 2.8 bc (T) | 55 d (D) | |
| LP | Low_Hill_W | 2.2 a (T) | 35 bc (D) | |
| TF | Coast_N | 3 a (D) | 76 ab (D) | |
| TF | Coast_S | 3.3 a (D) | 98 b (D) | |
| TF | Hill_N | 3.2 a (D) | 83 ab (D) | |
| TF | Hill_S | 3.2 a (D) | 90 a (D) | |
| TF | High | 3.1 a (D) | 95 a (D) | |
| TF | Low | 3.3 b (D) | 146 b (D) | |
| TF | High_Coast_N | 2.8 a (D) | 37 a (D) | |
| TF | High_Coast_S | 2.6 a (D) | 36 a (D) | |
| TF | High_Hill_N | 2.8 a (D) | 46 ab (D) | |
| TF | High_Hill_S | 3.1 ab (D) | 59 bc (D) | |
| TF | Low_Coast_N | 2.8 a (D) | 62 c (D) | |
| TF | Low_Coast_S | 3.2 b (D) | 89 d (D) | |
| TF | Low_Hill_N | 2.9 ab (D) | 59 bc (D) | |
| TF | Low_Hill_S | 3 ab (D) | 63 cd (D) | |

| Table S15 Details of retained GLMMs and model-selection support for fungal guild richness analyses | | | | | | | | | | |  |
| --- | --- | --- | --- | --- | --- | --- | --- | --- | --- | --- | --- |
| Island | Guild | Response | Family | Retained model | Random effect | AIC | Selection comparison | LR_chisq | Df | P_value | |
| TF | Sapro | Saprophytic genera (n) | Neg binom | Host condition + Zone | Location | 868.4 | Additive model retained by parsimony; interaction not supported | 7.22 | 3 | 0.065 | |
|  | Endo | Endophytic genera (n) | Poisson | Host condition + Zone | Location | 465.7 | Additive model retained by parsimony; interaction not supported | 1.40 | 3 | 0.705 | |
|  | Plant Patho | Plant pathogenic genera (n) | Poisson | Host condition + Zone | Location | 822.8 | Additive model retained by parsimony; interaction not supported | 3.34 | 3 | 0.342 | |
| LP | Sapro | Saprophytic genera (n) | Neg binom | Host condition * Zone | Location | 318.3 | Interaction model retained | 9.19 | 3 | 0.027 | |
|  | Endo | Endophytic genera (n) | Poisson | Host condition * Zone | Location | 187.99 | Interaction model retained | 10.64 | 3 | 0.014 | |
|  | Plant Patho | Plant pathogenic genera (n) | Poisson | Host condition + Zone | Location | 277.83 | Additive model retained by parsimony; interaction not supported | 1.50 | 3 | 0.681 | |

* AIC values refer to the retained model. LR_chisq, Df and P_value refer to the comparison used to evaluate whether a more complex alternative model should be retained. For rows with additive retained models, these statistics correspond to the comparison between the additive and interaction models; for rows with interaction retained models, they correspond to the comparison between the additive and interaction models supporting retention of the interaction.
